# β‐Elemene Rescues Radiation‐Induced Enteritis by Orchestrating a Host‐Microbiome Circuit That Fuels Epigenetic DNA Repair

**DOI:** 10.1002/advs.202521445

**Published:** 2026-05-27

**Authors:** Jiancheng He, Jiapeng Bao, Shukang Deng, Weijie Zang, Haoming Yan, Zihao Zhao, Guangze Zhang, Ruiqing Liu, Junjie Chen, Yilin Hu, Wanjiang Xue

**Affiliations:** ^1^ Department of Gastrointestinal Surgery Affiliated Hospital and Medical School of Nantong University Nantong China; ^2^ Department of General Surgery Binhai County People's Hospital Yancheng Jiangsu China; ^3^ Research Center of Clinical Medicine Affiliated Hospital of Nantong University Nantong China; ^4^ Nantong Key Laboratory of Gastrointestinal Oncology Nantong China

**Keywords:** DNA damage repair, gut microbiota, lactylation, radiation‐induced enteritis, β‐elemene

## Abstract

Radiation‐induced enteritis (RIE) is a severe, dose‐limiting toxicity of cancer radiotherapy lacking mechanism‐based therapies. While the gut microbiome regulates radiation injury, harnessing it therapeutically remains challenging. Here, we show that the natural product β‐elemene protects against RIE through a synergistic mechanism coordinating host and microbial responses. β‐elemene directly rescues the radiation‐disrupted interaction between the lactate transporter MCT1 and its chaperone CD147 in intestinal epithelial cells, priming them for enhanced lactate uptake. Concurrently, β‐elemene selectively enriches for *Lactobacillus gasseri*, increasing intestinal lactate production. The convergence of host priming and elevated lactate availability triggers a metabo‐epigenetic cascade. Specifically, lactate drives the lactylation of the chromatin‐associated protein RBBP4, which in turn recruits EP300 to activate the transcription of essential DNA damage repair genes. We further identify EP300 as a lactyl‐transferase, establishing a self‐amplifying positive feedback loop that robustly enhances the repair signal. Our findings delineate a complete drug‐microbe‐metabolite‐epigenome axis, establishing a ‘prime‐and‐fuel’ therapeutic strategy where a single agent orchestrates inter‐kingdom communication to promote tissue regeneration.

## Graphical abstract

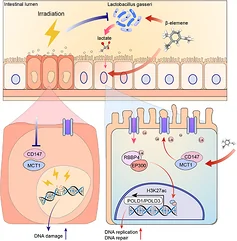

## 1 Introduction

The clinical application of radiotherapy, a cornerstone of therapy for abdominopelvic malignancies such as cervical, prostate, and rectal cancers, is frequently compromised by severe, dose‐limiting toxicities to adjacent healthy tissues [1]. Radiation‐induced enteritis (RIE) is a predominant complication, with up to 90% of patients experiencing acute gastrointestinal symptoms including diarrhea, nausea, and abdominal pain [2, 3]. These symptoms not only severely impair quality of life but can also necessitate treatment interruptions or dose reductions, potentially jeopardizing local tumor control [4]. The underlying pathophysiology involves acute mucosal inflammation and apoptosis of crypt stem cells, leading to villus atrophy and barrier dysfunction, which can progress to chronic complications like fibrosis and strictures [5]. Despite its high incidence and clinical impact, therapeutic options for RIE remain largely supportive, focusing on symptomatic relief rather than addressing the core mechanisms of mucosal injury and failed repair [6]. This highlights a critical unmet need for mechanism‐based interventions that can actively promote intestinal epithelial regeneration.

Emerging evidence implicates the gut microbiome as a pivotal regulator of the host's response to radiation [7, 8]. High‐energy ionizing radiation induces profound dysbiosis, typically characterized by a decreased abundance of beneficial Firmicutes and an overgrowth of pathobiont Proteobacteria [9]. This microbial imbalance is not a mere epiphenomenon but a core pathogenic driver that exacerbates mucosal injury by compromising intestinal barrier integrity, amplifying local inflammatory responses, and reducing the production of key protective metabolites, such as butyrate [8]. This has positioned the microbiome as a promising therapeutic target [10]. However, current strategies face significant hurdles. Fecal microbiota transplantation (FMT), while showing potential, is hampered by issues of standardization, engraftment variability, and safety concerns [11, 12]. Similarly, the efficacy of specific probiotics is often transient and strain‐dependent [13]. These limitations leave a fundamental question unresolved: can we move beyond broad‐spectrum microbial replacement and utilize small‐molecule drugs to precisely and robustly remodel the gut ecosystem to engage endogenous host repair programs?

In the search for such mechanism‐based interventions, natural products offer a rich source of bioactive compounds with highly favorable safety profiles [14]. β‐elemene, a naturally occurring sesquiterpenoid, stands out as a compelling candidate [15]. Unlike the only FDA‐approved radioprotectant, amifostine, whose clinical application is severely hampered by significant systemic toxicities such as hypotension, β‐elemene possesses a long history of safe clinical use with minimal normal‐tissue cytotoxicity [16, 17, 18, 19]. This exceptional safety window provides a strong foundational rationale for exploring its potential in mitigating normal tissue injury during radiotherapy. Furthermore, the mechanistic rationale for investigating its interplay with the gut microbiota stems from a modern pharmacological paradigm. Upon oral administration, natural sesquiterpenes frequently exhibit profound local gastrointestinal effects despite limited systemic bioavailability [20]. This suggests that their primary therapeutic targets may not solely be host cells, but rather the complex microbial consortium residing in the gut lumen. Given that high‐energy irradiation drastically disrupts this delicate ecosystem, it is a highly logical and necessary progression to investigate whether the gut microbiota serves as the primary sensory interface and indispensable mediator for the therapeutic efficacy of orally administered β‐elemene against RIE.

Here, we hypothesize that the gut microbiome is an indispensable mediator of β‐elemene's radioprotective efficacy. We posit that its primary mechanism involves modulating the gut ecosystem to generate therapeutic signals that activate host repair pathways, rather than acting through direct, isolated radioprotective effects on host cells. Using an integrated approach combining in vivo mouse models of RIE, microbiome and metabolome profiling, and in vitro cellular systems, this study aims to systematically test this hypothesis and elucidate the potential microbe‐dependent mechanism of action. This research seeks to provide a new therapeutic paradigm for RIE, based on a drug‐microbiota‐host signaling axis, and to lay the crucial theoretical and experimental foundation for a new generation of microbiome‐targeted radioprotectants.

## 2 Method

### 2.1 Animal Models and Treatments

RIE Model: Male C57BL/6J mice, 6–8 weeks old, were obtained from the Experimental Animal Center of Nantong University. All mice were housed in a specific pathogen‐free environment with ad libitum access to food and water. Following a 1‐week acclimatization period, the mice were divided into Cohort 1 for pathological observation and Cohort 2 for survival rate observation. Both cohorts were randomly assigned to one of four groups (n = 10/group): (1) PBS control group (PBS); (2) β‐elemene treatment group (β‐elemene); (3) Radiation + PBS group (IR+PBS); and (4) Radiation + β‐elemene group (IR+β‐elemene). Mice in the IR+PBS and IR+β‐elemene groups were subjected to a single 12 Gy dose of abdominal irradiation, delivered at a dose rate of 1 Gy/min. During the procedure, customized lead shields were utilized to protect the head and thorax [21]. Starting 7 days prior to irradiation (Day‐7), β‐elemene, dissolved in corn oil, was administered to the mice via oral gavage at a dose of 50 mg/kg once daily. β‐elemene was purchased from MedChemExpress (MCE, Monmouth Junction, NJ, USA). The body weight and survival status of the mice were monitored daily. At the experimental endpoints (Cohort 1: Day 7; Cohort 2: Day 21), the mice were euthanized, and the contents of the small intestine and cecum were collected for subsequent analysis.

Gut Microbiota Depletion Model: To investigate the role of gut microbiota, a broad‐spectrum antibiotic cocktail (ABX) was used to deplete the gut microbiota in mice before establishing the RIE model. Subsequently, the RIE model was established, and β‐elemene intervention was performed as described above. The mice were divided into Cohort 3 for pathological observation and Cohort 4 for survival rate observation. Both cohorts were randomly assigned to one of four groups (n = 10/group): (1) Radiation + PBS control group (IR+PBS); (2) Radiation + β‐elemene treatment group (IR+β‐elemene); (3) Antibiotics + Radiation + PBS group (ABX+IR+PBS); and (4) Antibiotics + Radiation + β‐elemene group (ABX+IR+β‐elemene).

Germ‐Free (GF) Mouse Model: Germ‐free C57BL/6J mice were maintained in positive‐pressure sterile isolators. The mice were divided into Cohort 5 for pathological observation and Cohort 6 for survival rate observation. The GF mice were randomly assigned to one of four groups (n = 10/group): (1) IR+PBS; (2) IR+*L. gasseri (Lactobacillus gasseri)* colonization; (3) IR+β‐elemene; and (4) IR+*L. gasseri* colonization + β‐elemene (Combined). 7 days prior to irradiation, mice in the corresponding groups were colonized with *L. gasseri* ([BeNa Culture Collection, BNCC135322], at a daily dose of 1 × 10^9^ CFU/mouse for three consecutive days) via oral gavage. Subsequently, abdominal irradiation and β‐elemene treatment were performed as described above. To specifically address the necessity of β‐elemene‐mediated priming for lactate utilization in vivo, an independent supplemental experiment was performed using additional cohorts of GF C57BL/6J mice (Cohort 7 for pathology and Cohort 8 for survival; n = 40/cohort).Mice were randomly assigned to one of four groups (n = 10/group): (1) IR+PBS; (2) IR+Lactate; (3) IR+*L. gasseri*+Lactate; and (4) IR+β‐elemene+Lactate. Sodium lactate (Sigma‐Aldrich) was administered via oral gavage (1 g/kg, once daily) starting 7 days prior to irradiation and continuing until the experimental endpoint (day 7 post‐irradiation). The dosing and irradiation protocols remained identical to those described above.

The Animal Experimentation and Ethics Committee of Nantong University granted approval for all animal‐related studies (P20230214‐002), and the procedures adhered to contemporary standards for animal well‐being and care.

### 2.2 Histology and Immunohistochemistry (IHC)

Tissue samples from the terminal ileum of the mice were fixed in 4% paraformaldehyde for 24 h, embedded in paraffin, and sectioned into 4 µm‐thick slices. Hematoxylin and Eosin (H&E) staining was performed to assess histopathological changes. Villus height and crypt depth were measured using ImageJ software. Evaluation of H&E‐stained sections was performed by two independent pathologists who were blinded to the experimental groups. Histopathological changes were graded on a scale of 0 to 5 based on the criteria established by the Chiu's method [22]: Grade 0, normal intestinal mucosal villi; Grade 1, sub‐epithelial capillary congestion and cystic spaces below the villus tip; Grade 2, enlarged sub‐epithelial cystic spaces, diffuse edema in the lamina propria, and dilation of central lacteals; Grade 3, degeneration and necrosis of intestinal epithelial cells, severe edema in the lamina propria, with occasional sloughing at the villus tips; Grade 4, degeneration, necrosis, and sloughing of intestinal epithelial cells, capillary congestion and dilation, exposure of the lamina propria, and partial villus denudation; Grade 5, hemorrhage, ulceration, disintegration of the lamina propria, and villus denudation. In cases of disagreement between the two experts, a joint review was conducted under a high‐power microscope. If necessary, a third expert was invited for re‐evaluation to reach a final conclusion.

For IHC, paraffin sections underwent dewaxing, rehydration, and antigen retrieval, followed by blocking of endogenous peroxidase with 3% H_2_O_2_. After blocking with 5% BSA, the sections were incubated overnight at 4°C with primary antibodies, including: ZO‐1 (Proteintech, 21773‐1‐AP) and Occludin (Proteintech, 27260‐1‐AP). The following day, sections were incubated with an HRP‐labeled secondary antibody, visualized with DAB, and counterstained with hematoxylin.

### 2.3 Immunofluorescence and TUNEL Analysis

For Immunofluorescence staining, paraffin sections were processed similarly to IHC. Primary antibodies included: Ki‐67 (Proteintech, 27309‐1‐AP), Cleaved‐Caspase 3 (Proteintech, 25128‐1‐AP), MCT1 (Invitrogen, MA5‐18288), CD147 (Proteintech, 11989‐1‐AP), γH2AX (CST, 2577S), and RAD51 (Proteintech, 4961‐1‐AP). Subsequently, sections were incubated with corresponding Alexa Fluor‐conjugated secondary antibodies (Invitrogen), and nuclei were counterstained with DAPI.

Cell apoptosis was detected using the Terminal deoxynucleotidyl transferase dUTP nick end labeling (TUNEL) assay kit (Beyotime, C1091), following the manufacturer's protocol strictly. All images were captured using a laser scanning confocal microscope (ZEISS, LSM 900). Multiplex Immunohistochemistry (mIHC) was conducted using the mIHC staining kit (absin, Shanghai, China) following the manufacturer's instructions. Staining was performed sequentially for MCT1 (Invitrogen, MA5‐18288), CD147 (Proteintech, 11989‐1‐AP), RBBP4‐K26la, POLD1 (Proteintech, 15646‐1‐AP), and POLD3 (Proteintech, 21935‐1‐AP).

### 2.4 16S rRNA Gene Sequencing

Genomic DNA was extracted from samples using the CTAB or SDS method, and the purity and concentration of the DNA were assessed. The V3‐V4 variable region was amplified by PCR using specific primers with barcodes and high‐fidelity DNA polymerase. The PCR products were verified by 2% agarose gel electrophoresis, and the target fragments were recovered using the AxyPrep DNA Gel Extraction Kit (Axygen). Based on the preliminary quantification from electrophoresis, the recovered PCR products were quantified using the QuantiFluor‐ST Blue Fluorescence Quantitative System (Promega). The samples were then mixed in proportions corresponding to the required sequencing volume for each. Library preparation was conducted using the NEBNext Ultra DNA Library Prep Kit. The quality of the constructed libraries was assessed using an Agilent Bioanalyzer 2100 and Qubit. Qualified libraries were then sequenced. Principal Coordinate Analysis (PCoA) based on weighted UniFrac distances was performed to evaluate beta diversity. Alpha diversity was calculated using the Shannon index. Linear discriminant analysis Effect Size (LEfSe) and STAMP software were used to identify significantly different microbial taxa between groups.

### 2.5 Non‐Targeted Metabolomics Analysis

Mouse ileocecal content samples were slowly thawed at 4°C. An appropriate amount of each sample was added to a pre‐chilled methanol/acetonitrile/water solution (2:2:1, v/v), vortexed, ultrasonicated at low temperature for 30 min, and incubated at −20°C for 10 min. The mixture was then centrifuged at 14 000 g for 20 min at 4°C. The supernatant was collected and dried under vacuum. For mass spectrometry analysis, the dried residue was reconstituted in 100 µL of an acetonitrile‐water solution (1:1, v/v), vortexed, and centrifuged at 14 000 g for 15 min at 4°C. The resulting supernatant was injected for analysis. The raw data were converted to. mzXML format using ProteoWizard, and then processed with XCMS software for peak alignment, retention time correction, and peak area extraction. The data extracted by XCMS underwent metabolite identification and data preprocessing, followed by an evaluation of experimental data quality, and finally, data analysis. Partial Least Squares Discriminant Analysis (PLS‐DA) was used to identify differences in the metabolic profiles between groups.

### 2.6 Cell Culture and Treatments

The human small intestinal epithelial cell line HIEC‐6 was purchased from YaJi Biological (YS3102C) and cultured in DMEM supplemented with 10% fetal bovine serum and 1% penicillin/streptomycin at 37°C in a 5% CO_2_ atmosphere. Cells were subjected to 4 Gy or 8 Gy of X‐ray irradiation. β‐elemene and lactate (Sigma‐Aldrich) were added to the culture medium immediately after irradiation at concentrations determined by the experimental design. The EP300 inhibitor C646 and agonist CTB were purchased from MedChemExpress.

### 2.7 Cell Viability and Apoptosis Assays

Cell viability was assessed using the Cell Counting Kit‐8 (CCK‐8, Dojindo). Cells were seeded in 96‐well plates, and after 24 h of treatment, 10 µL of CCK‐8 solution was added to each well. After a 2‐h incubation, the absorbance was measured at 450 nm. Cell apoptosis was detected using the Annexin V‐FITC/PI Apoptosis Detection Kit (BD Biosciences). After treatment, cells were collected, stained according to the manufacturer's instructions, and analyzed by a flow cytometer (Beckman, DxFLEX).

### 2.8 RNA Sequencing Analysis (RNA‐Seq)

We conducted mRNA sequencing on HIEC‐6 cells, using the Illumina sequencing platform provided by Gene Denovo Biotechnology Co., Ltd (Guangzhou, China).

### 2.9 Real‐Time Quantitative PCR (RT‐qPCR)

Total RNA was extracted using TRIzol reagent (Invitrogen). The RNA was reverse transcribed into cDNA using the HiScript III RT SuperMix for qPCR (+gDNA wiper) (Vazyme, R323‐01). The qPCR reaction was performed using ChamQ Universal SYBR qPCR Master Mix (Vazyme, Q711‐02) on a QuantStudio 5 system (Thermo Fisher, A28575). Relative gene expression was calculated using the 2‐ΔΔCt method. Primer sequences are listed in Table S1.

### 2.10 Western Blot and Co‐Immunoprecipitation (Co‐IP)

Total protein was extracted using RIPA lysis buffer and quantified by the BCA method. Subcellular fractions (membrane and cytoplasmic proteins) were extracted using the Membrane and Cytosol Protein Extraction Kit (Beyotime, P0033). Proteins were separated by SDS‐PAGE and transferred to PVDF membranes. The membranes were blocked with 5% non‐fat milk and incubated with primary antibodies overnight at 4°C. Information on primary antibodies is available in Table S2. The next day, membranes were incubated with HRP‐conjugated secondary antibodies for 1 h at room temperature and visualized using an ECL chemiluminescence kit.

For Co‐IP, cells were treated with a pre‐chilled non‐denaturing IP lysis buffer supplemented with protease and phosphatase inhibitors. The cleared protein supernatant was collected after high‐speed centrifugation and quantified by BCA. Equal amounts of protein lysate were incubated with either a specific primary antibody or an isotype‐matched IgG control, rotating overnight at 4°C to form immunocomplexes. Subsequently, Protein A/G magnetic beads (Santa Cruz Biotechnology, sc‐2003) were added and incubated with rotation for 2–4 h to capture the complexes. The beads were then washed thoroughly at least four times with IP lysis buffer to remove all non‐specifically bound proteins. Finally, the bound proteins were eluted by adding 2X SDS‐PAGE loading buffer and heating at 95°C–100°C for 10 min. The supernatant was collected after magnetic separation and analyzed by Western Blot alongside the Input (total lysate) control. Full and unaltered original images of all Western blots presented in this study are provided in Figures S8–S12.

### 2.11 siRNA Knockdown and Plasmid Transfection

siRNAs targeting *POLD1*, *POLD3*, *EP300*, *CD147*, and *RBBP4*, as well as a negative control siRNA, were purchased from GeneAdv Co. Ltd (Suzhou, China). The sequences are listed in Table S3. Plasmids for Flag‐MCT1, HA‐CD147, and Flag‐RBBP4 (wild‐type and K26R, K114R, K120R, K212R, K220R, K309R mutants) were constructed by Gene Adv (Huzhou, China). Plasmid and siRNA transfection were conducted using Polyplus jetPRIME transfection reagent (Polyplus, France) in a six‐well plate, following the manufacturer's instructions.

### 2.12 Comet Assay

The Comet Assay was performed strictly following the manufacturer's instructions for the OxiSelect 96‐Well Comet Assay Kit (CELL BIOLABS, STA‐355). Briefly, cells were prepared as a single‐cell suspension at a concentration of 1 × 10^5^ cells/mL and mixed with pre‐melted Comet Agarose (maintained at 37°C) at a 1:10 volume ratio. A 20 µL aliquot of this mixture was immediately added to a 96‐Well Comet Slide and allowed to solidify by incubating at 4°C in the dark for 15 min. The slide was then immersed in pre‐chilled Lysis Buffer and incubated at 4°C in the dark for 30–60 min. After lysis, the buffer was replaced with a pre‐chilled Alkaline Solution for an additional 30‐min incubation at 4°C in the dark to complete DNA denaturation and unwinding. Electrophoresis was performed in a horizontal chamber with pre‐chilled alkaline electrophoresis buffer at 1 volt/cm and 300 mA for 15–30 min. After electrophoresis, the slide was washed sequentially with deionized water and 70% ethanol, and then completely air‐dried. Finally, 50 µL of diluted Vista Green DNA Dye was added to each well, incubated for 15 min at room temperature in the dark, and visualized for image acquisition using a fluorescence microscope equipped with a FITC filter.

### 2.13 Chromatin Immunoprecipitation (ChIP)‐qPCR

ChIP was performed using the SimpleChIP Plus Sonication Chromatin IP Kit (CST, #56383). Cells were cross‐linked with 1% formaldehyde, and chromatin was fragmented by sonication or enzymatic digestion. DNA‐protein complexes were immunoprecipitated using specific antibodies (H3K27ac, H3K4me3, H3K4me1, H3K4me2, H3K18la, H3K9la) or an IgG control. After reversing the cross‐links, the DNA was purified and analyzed by qPCR to quantify the promoter regions of *POLD1* and *POLD3*. Primer sequences and antibodies are listed in Table S4.

### 2.14 Bioinformatics Analysis

AlphaFold2 was used to predict the protein interaction structures of MCT1 with CD147 and EP300 with RBBP4 [23]. The Cistrome Data Browser [24] and GTRD databases [25] were used to predict histone modifications and transcriptional regulatory factors for *POLD1* and *POLD3*. Gene expression correlation analysis was performed based on the GEPIA2 database [26].

### 2.15 Immunoprecipitation and Mass Spectrometry Analysis

Cells from each sample (3 × 10^7^ cells) were lysed in 1.2 mL of RIPA buffer for 30 min in separate reactions. For each immunoprecipitation, 1 mg of total protein (normalized to a 1 mL volume with IP lysis buffer) was incubated with 5 µg of pan‐lactylation antibody (or an equivalent amount of rabbit IgG as a control) and 40 µL of protein A/G agarose beads overnight at 4°C. Then, the beads were washed repeatedly with pre‐cold lysis buffer and centrifuged at 1000 rpm, 4°C for 5 min. An appropriate amount of TEAB was added to adjust pH 8.0.5 µL of suspension was used for SDS‐PAGE testing. For digestion, the protein solution was reduced with 5 mM dithiothreitol for 30 min at 56°C and alkylated with 11 mM iodoacetamide for 15 min at room temperature in darkness. The protein sample was then diluted by adding 100 mM TEAB to urea concentration less than 2 M. Finally, trypsin was added at 1:50 trypsin‐to‐protein mass ratio for the first digestion overnight and 1:100 trypsin‐to‐protein mass ratio for a second 4 h‐digestion. Finally, the peptides were desalted by C18 SPE column. The tryptic peptides were dissolved in solvent A, directly loaded onto a home‐made reversed‐phase analytical column (25‐cm length, 100 um i.d.). The mobile phase consisted of solvent A (0.1% formic acid, 2% acetonitrile/in water) and solvent B (0.1% formic acid in acetonitrile). Peptides were separated with following gradient: 0–14 min, 6%–24%B;14‐16 min, 24%–35%B; 16–18 min, 35%–80%B; 18–20 min, 80%B, and all at a constant flow rate of 500 nL/min on a NanoElute UHPLC system (Bruker Daltonics). The peptides were subjected to capillary source followed by the timsTOF Pro mass spectrometry. The electrospray voltage applied was 1.75 KV. Precursors and fragments were analyzed at the TOF detector. The timsTOF Pro was operated in data independent parallel accumulation serial fragmentation (dia‐PASEF) mode. The full MS scan was set as 300–1500 (MS/MS scan range) and 20PASEF (MS/MS mode) ‐MS/MS scans were acquired per cycle. The MS/MS scan range was set as 400–850 and isolation window was set as 7 m/z. The DIA data were processed using DIA‐NN search engine (v.1.8). Tandem mass spectra were searched against the Homo_sapiens 9606_SP_20241202.fasta (20422 entries) concatenated with reverse decoy database. Trypsin/P was specified as cleavage enzyme allowing up to 1 missed cleavages. Excision on N‐term Met and carbamidomethyl on Cys were specified as fixed modification. FDR was adjusted to <1%.

### 2.16 Statistical Analysis

Data were analyzed using GraphPad Prism 10.0 and the R programming language. Measurement data with a normal distribution were expressed as mean ± SD and statistically analyzed with a two‐sample t‐test. Non‐normal distribution measurement data were expressed as median (range) and analyzed by the Mann‐Whitney U test. For groups of three or more that conform to a normal distribution, one‐way ANOVA is utilized, while for those that do not follow a normal distribution, the Kruskal–Wallis test is employed. Survival rates were calculated by Log‐rank analysis and Kaplan‐Meier. All statistical analyses were two‐sided, and p < 0.05 was used to define statistical significance. All experiments were conducted more than three times.

## 3 Results

### 3.1 β‐elemene Drives Intestinal Epithelial Regeneration to Protect Against Radiation‐Induced Injury

**FIGURE 1:**
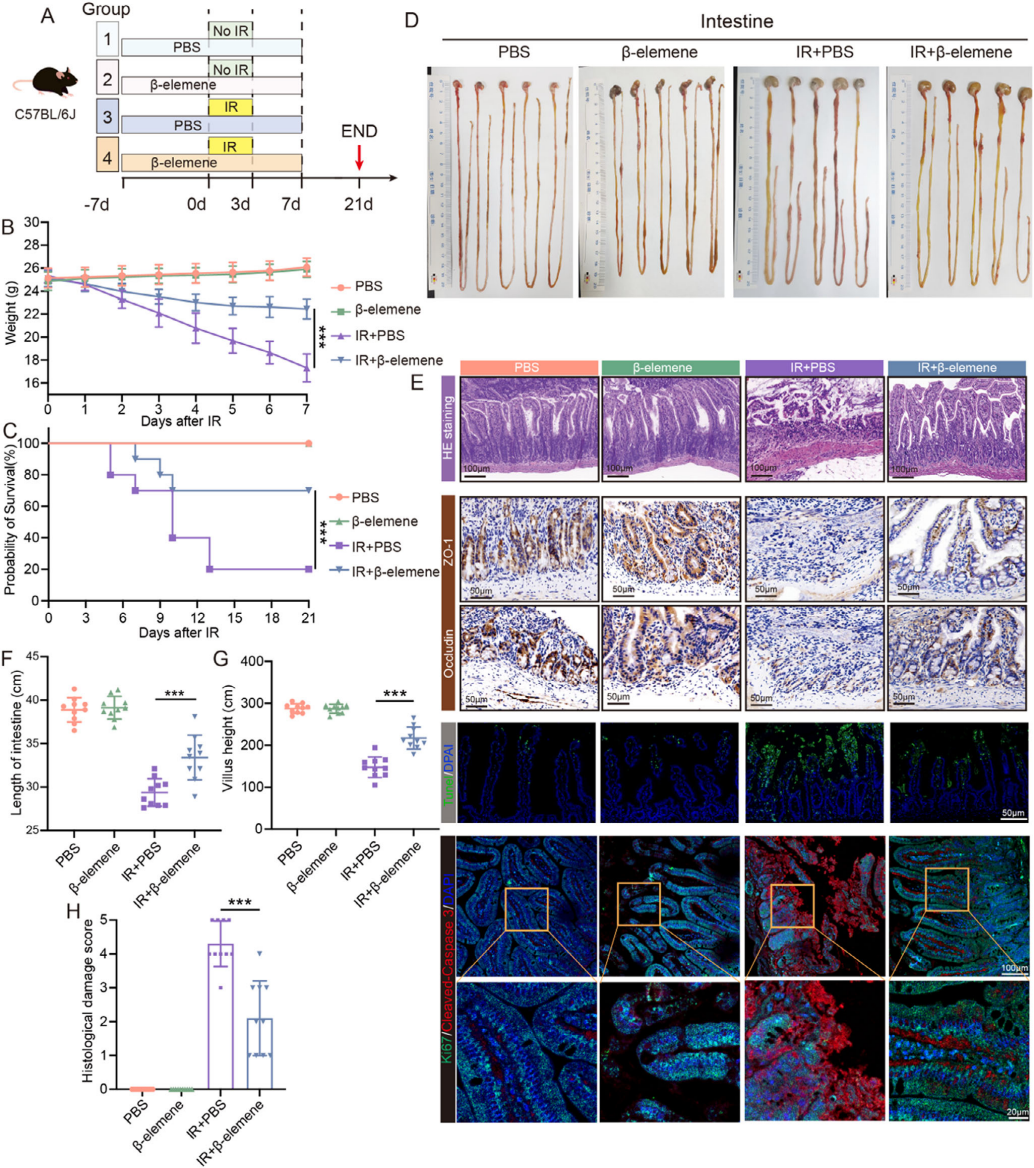
β‐elemene Drives Intestinal Epithelial Regeneration to Protect Against Radiation‐induced Injury (A) Schematic illustration of the experimental design for the radiation‐induced enteritis model in C57BL/6J mice. Mice were divided into four groups: a PBS control group (PBS), a β‐elemene treatment group (β‐elemene), a radiation+PBS group (IR+PBS), and a radiation+β‐elemene group (IR+β‐elemene). (B) Body weight change curves for each group of mice within 7 days after abdominal irradiation (IR). (C) Kaplan‐Meier survival curves for each group of mice within 21 days after IR. (D) Representative images of the gross morphology of small intestines from each group at the experimental endpoint. (E) Representative histological images of ileal tissues from each group. From top to bottom: Hematoxylin and Eosin (H&E) staining (scale bar = 100 µm); immunohistochemical (IHC) staining for the tight junction proteins ZO‐1 and Occludin (scale bar = 50 µm); TUNEL staining for apoptosis (green) (scale bar = 50 µm); and immunofluorescence (IF) co‐staining for the proliferation marker Ki‐67 (green) and the apoptosis marker Cleaved‐Caspase 3 (red), with nuclei counterstained with DAPI (blue) (scale bar = 100 µm). (F–H) Quantification of small intestine length (F), villus height (G), and histological damage score (H). All data are presented as mean ± standard deviation (SD). *** *p* < 0.001.

To determine if β‐elemene could protect against radiation‐induced intestinal damage in vivo, we subjected mice to abdominal irradiation (Figure 1A). Two independent cohorts were established to comprehensively evaluate the therapeutic effect: Cohort 1 was dedicated to pathological analysis (n = 40), while Cohort 2 was monitored for long‐term survival (n = 40).

Strikingly, β‐elemene treatment not only mitigated radiation‐induced weight loss but also robustly increased the overall survival of the animals compared to vehicle controls (Figure 1B,C). Findings from Cohort 1 revealed that this systemic protection was reflected at the organ level, where β‐elemene prevented the significant intestinal shortening and mucosal atrophy caused by irradiation (Figure 1D,E). Histological examination confirmed that β‐elemene preserved the integrity of the villus‐crypt architecture, resulting in significantly taller villi and dramatically reduced tissue damage scores (Figure 1F–H). We next sought to uncover the cellular basis for this protection. A key aspect of intestinal homeostasis is the epithelial barrier, and we found that β‐elemene maintained this barrier by preventing the radiation‐induced loss of the tight junction proteins Zonula occludens‐1 (ZO‐1) and Occludin (Figure 1E). Critically, the regenerative capacity of the epithelium depends on a fine‐tuned balance between cell proliferation and apoptosis. We discovered that β‐elemene powerfully shifts this balance toward regeneration. Immunofluorescence analysis revealed a surge in the proliferation marker Ki‐67 alongside a sharp decrease in the apoptotic effector Cleaved‐Caspase 3 in the intestinal crypts of β‐elemene‐treated mice (Figure 1E). This anti‐apoptotic effect was substantiated by a decrease in TUNEL‐positive cells (Figure 1E).

Thus, our in vivo data establish β‐elemene as a potent protective agent that promotes intestinal tissue repair and animal survival post‐irradiation by bolstering the epithelial barrier and decisively promoting a pro‐regenerative program of enhanced cell proliferation and suppressed apoptosis.

### 3.2 The Radioprotective Effect of β‐elemene Is Abrogated by Microbiota Depletion

**FIGURE 2:**
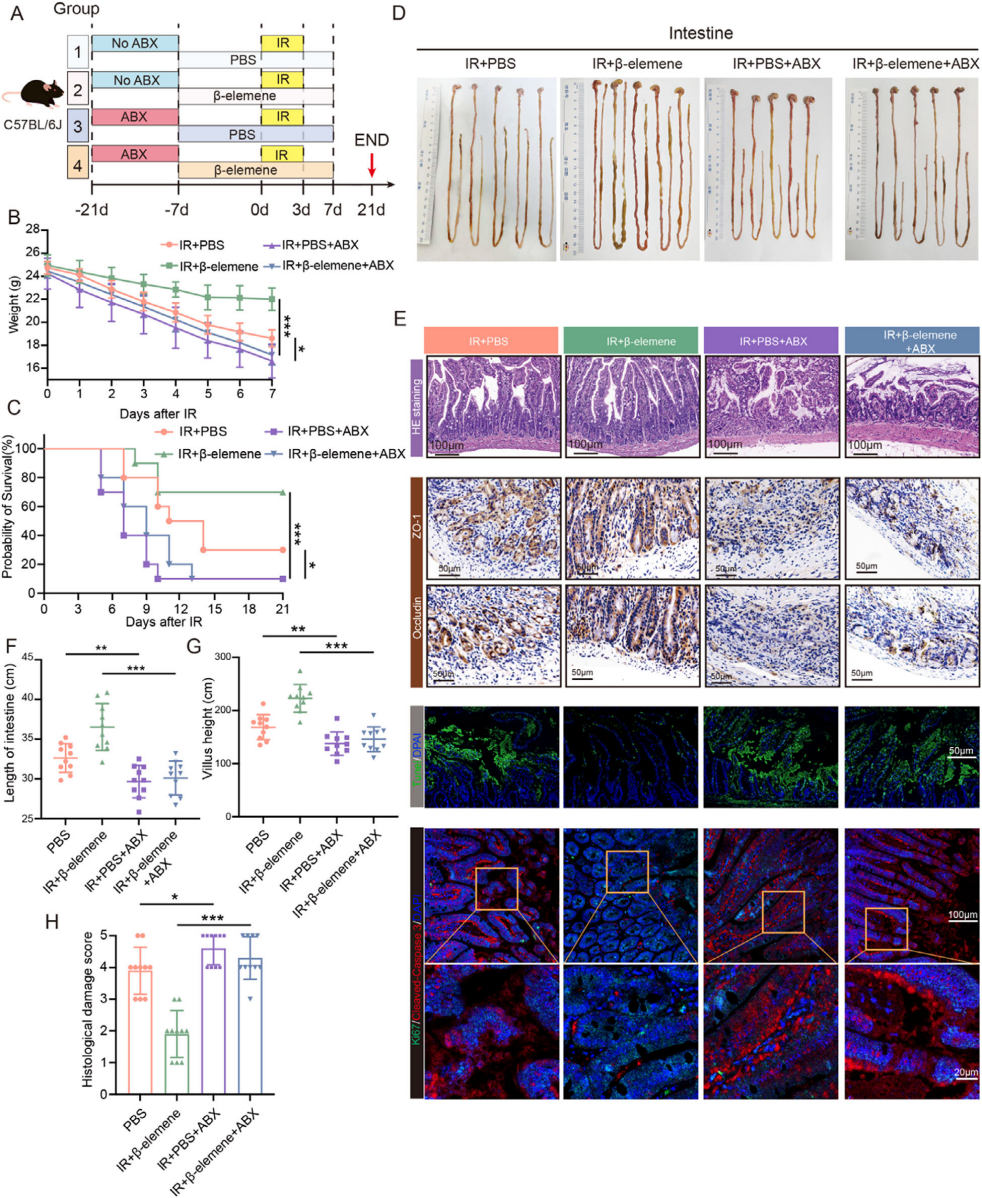
The Radioprotective Effect of β‐elemene Is Abrogated by Microbiota Depletion (A) Schematic illustration of the experimental design involving the depletion of gut microbiota in mice via an antibiotic cocktail (ABX) treatment. (B) Body weight change curves for mice, with or without ABX pretreatment, within 7 days after abdominal irradiation (IR). (C) Kaplan‐Meier survival curves for each group of mice within 21 days after IR. (D) Representative images of the gross morphology of small intestines from each group at the experimental endpoint. (E) Representative histological images of ileal tissues from each group. From top to bottom: H&E staining (scale bar = 100 µm); IHC staining for the tight junction proteins ZO‐1 and Occludin (scale bar = 50 µm); TUNEL staining for apoptosis (green); and IF co‐staining for the proliferation marker Ki‐67 (green) and the apoptosis marker Cleaved‐Caspase 3 (red), with nuclei counterstained with DAPI (blue) (scale bar = 100 µm). (F–H) Quantification of small intestine length (F), villus height (G), and histological damage score (H). All data are presented as mean ± SD. Statistical significance was assessed by appropriate statistical tests. *** *p* < 0.001.

Given the pivotal role of the gut microbiota in host homeostasis and injury response [27], we sought to determine whether the efficacy of β‐elemene was microbiota‐dependent. To test this, we depleted the gut microbiota using an antibiotic cocktail (ABX) prior to irradiation and drug intervention (Figure 2A). The mice were similarly allocated to two cohorts for pathological analysis (Cohort 3, n = 40) and survival observation (Cohort 4, n = 40).

In mice pre‐treated with ABX, the protective effects of β‐elemene were completely lost. The treatment failed to mitigate body weight loss, improve survival rates, or prevent the shortening of the small intestine (Figure 2B–D).

This conclusion was further supported by histological analysis. In the context of microbiota depletion, the improvements in villus height and tissue damage scores previously conferred by β‐elemene were no longer observed (Figure 2E–G). Likewise, the ability of β‐elemene to maintain the expression of tight junction proteins ZO‐1 and Occludin was eliminated. Mechanistically, microbiota depletion nullified the pro‐regenerative effects of β‐elemene, as it no longer promoted epithelial proliferation or inhibited apoptosis (Figure 2E). Taken together, these data unequivocally demonstrate that the therapeutic efficacy of β‐elemene against radiation‐induced enteritis is critically dependent on an intact gut microbiota.

### 3.3 β‐elemene Remodels the Microbiota to Promote a Radioprotective L. Gasseri‐Lactate Axis

**FIGURE 3:**
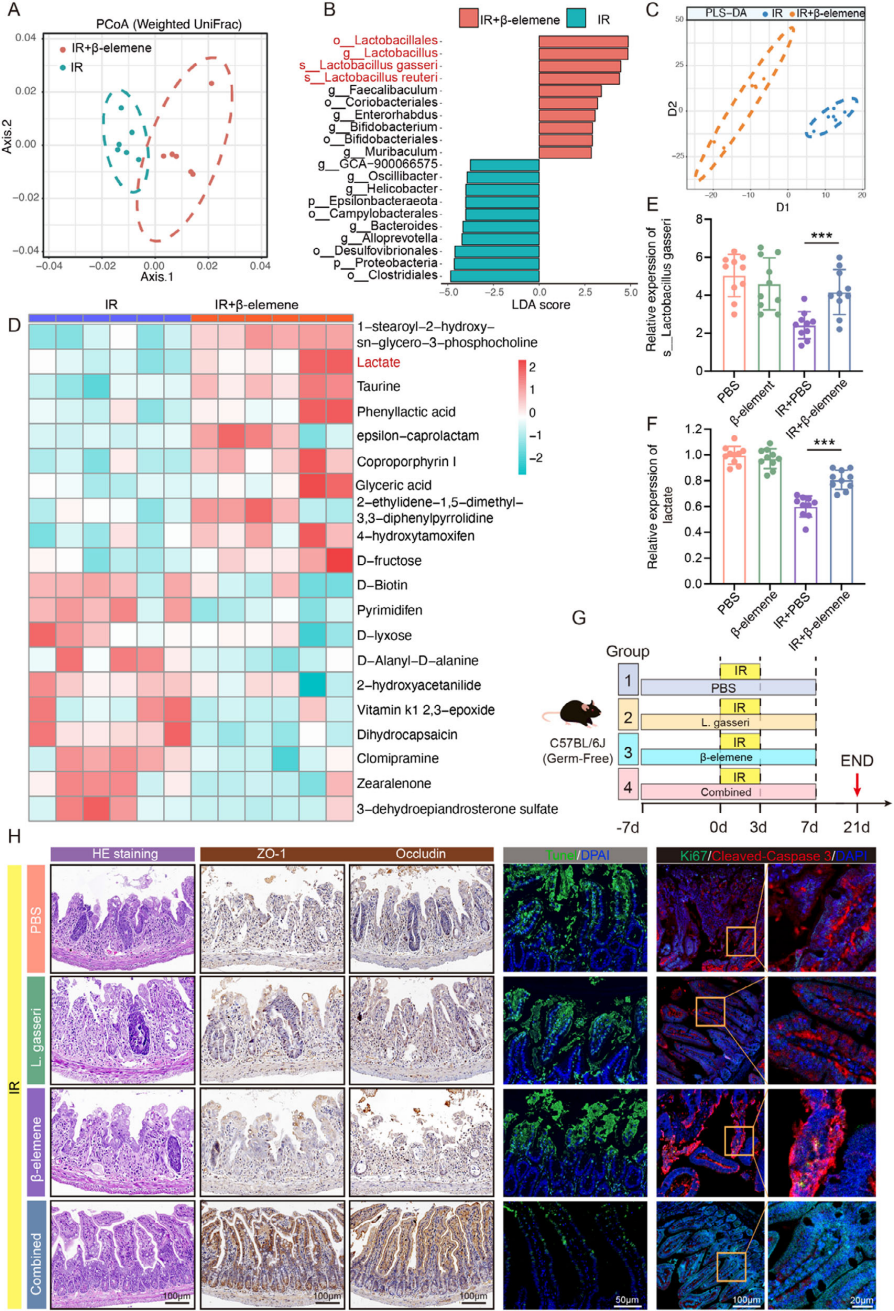
β‐elemene Remodels the Microbiota to Promote a Radioprotective *L. gasseri*‐lactate Axis (A) Principal Coordinates Analysis (PCoA) of β‐diversity based on weighted UniFrac distances, showing a significant difference in gut microbiota structure between the abdominal irradiation (IR) group and the IR+β‐elemene group. (B) Linear discriminant analysis Effect Size (LEfSe) plot showing bacterial taxa significantly enriched in the IR+β‐elemene group, with *L. gasseri* identified as a key biomarker. (C) Partial Least Squares Discriminant Analysis (PLS‐DA) plot based on non‐targeted metabolomics data from cecal contents. (D) Heatmap displaying significantly different metabolites between the IR and IR+β‐elemene groups. (E‐F) Validation by RT‐qPCR and lactate concentration measurement, respectively, confirming that β‐elemene treatment significantly increased the abundance of *L. gasseri* (E) and the concentration of lactate (F) in the intestine of the initial animal model (Figure 1). (G) Schematic illustration of the colonization experiment in germ‐free (GF) mice, designed to validate the causal role of *L. gasseri* in β‐elemene‐mediated radioprotection. (H) Representative histological images of ileal tissues from each group in the GF mouse experiment. From left to right: H&E staining (scale bar = 100 µm); IHC staining for the tight junction proteins ZO‐1 and Occludin (scale bar = 50 µm); TUNEL staining for apoptosis (green); and IF co‐staining for the proliferation marker Ki‐67 (green) and the apoptosis marker Cleaved‐Caspase 3 (red), with nuclei counterstained with DAPI (blue) (scale bar = 100 µm). All data are presented as mean ± SD. Statistical significance was assessed by appropriate statistical tests. *** *p* < 0.001.

Having established that the gut microbiota is indispensable for β‐elemene's radioprotective action, we aimed to pinpoint the specific microbial players. We profiled the cecal microbiome using 16S rRNA sequencing (Figure S1A). β‐elemene induced a specific and significant shift in community composition (β‐diversity) without altering overall diversity (α‐diversity), indicating a targeted effect (Figure 3A; Figure S1B,C). LEfSe analysis, independently confirmed by STAMP, unequivocally identified a single species, *L. gasseri*, as the top biomarker and most enriched taxon in β‐elemene‐treated mice (Figure 3B; Figure S1D;Tables S5–S6).

This striking enrichment prompted us to investigate the functional metabolic consequences. Untargeted metabolomics revealed a distinct metabolic landscape (Figure 3C), and lactate was identified as one of the most highly elevated metabolites, creating a direct link between our genomic and metabolomic findings (Figure 3D, Table S7). To connect these molecular changes directly to the observed phenotype, we performed targeted validation on cecal samples from Cohort 1 animals. This confirmed that β‐elemene treatment specifically restored both *L. gasseri* abundance and lactate levels in mice that had shown improved pathological outcomes (Figure 3E,F). Interestingly, correlation analysis among the abundance of *L. gasseri*, lactate concentration, and histological damage score revealed several key relationships. The abundance of *L. gasseri* was significantly and positively correlated with lactate concentration (R = 0.636, p < 0.001). Conversely, *L. gasseri* abundance was negatively correlated with the histological damage score (R = −0.656, p < 0.001). Similarly, lactate concentration was also strongly and negatively correlated with the histological damage score (R = −0.850, p < 0.001) (Figure S1E). Furthermore, our in vitro kinetic assay directly confirmed the robust lactate‐producing capacity of L. gasseri (Figure S1F).

These data established a strong correlative *L. gasseri*‐lactate axis, which we then tested for causality using two cohorts of germ‐free mice (Cohorts 5 and 6, Figure 3G). As hypothesized, neither β‐elemene nor *L. gasseri* alone conferred protection. In contrast, the combination of β‐elemene with *L. gasseri* colonization fully restored radioprotection (Figure 3H; Figure S2A–H). Intriguingly, β‐elemene enhanced the fitness of the colonizing bacterium, as mice receiving the combination displayed significantly higher loads of *L. gasseri* and lactate than mice receiving the bacterium alone (Figure S2A–H). Together, these experiments functionally establish that β‐elemene requires *L. gasseri* to exert its protective effects, and that this partnership is central to the therapeutic mechanism.

To further verify that the radioprotective effect requires both the metabolic substrate (lactate) and the drug‐induced uptake machinery, We conducted an additional supplemental experiment using GF mice treated with lactate alone or a combination of *L. gasseri* and lactate (Figure S3A). Strikingly, in the absence of β‐elemene, neither intervention could mitigate radiation‐induced weight loss, intestinal shortening, or mucosal damage (Figure S3B–J), despite achieving high intestinal lactate concentrations (Figure S3I). These data unequivocally establish that β‐elemene‐mediated host ‘priming’ is the indispensable rate‐limiting step for the therapeutic efficacy of microbial lactate.

### 3.4 A Synergistic Partnership Between β‐elemene and Lactate Protects Epithelial Cells From Radiation Injury

Our discovery of a *L. gasseri*‐lactate axis in vivo prompted us to ask whether β‐elemene and lactate interact directly at the cellular level to confer protection. To test this, we moved to an in vitro system using HIEC‐6 intestinal epithelial cells exposed to radiation (4 or 8 Gy).

**FIGURE 4:**
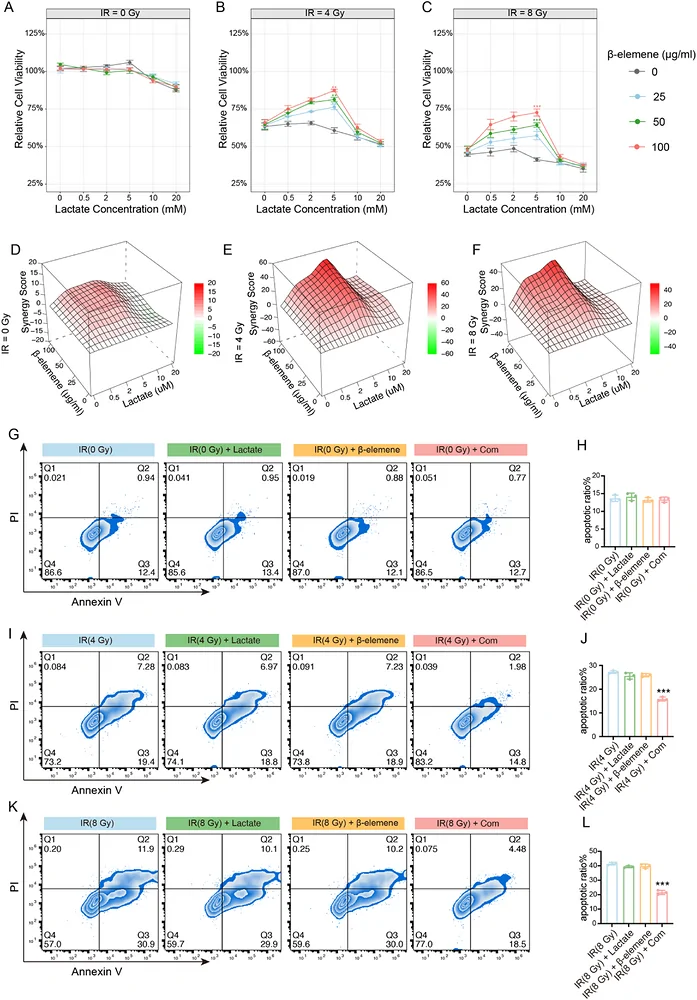
A Synergistic Partnership Between β‐elemene and Lactate Protects Epithelial Cells From Radiation Injury (A‐C) The effect of different concentrations of β‐elemene and lactate on the relative viability of the human intestinal epithelial cell line (HIEC‐6) under 0 Gy (A), 4 Gy (B), or 8 Gy (C) X‐ray irradiation (IR) conditions. (D‐F) Three‐dimensional synergy score plots for the effects of β‐elemene and lactate on HIEC‐6 cell viability at irradiation doses of 0 Gy (D), 4 Gy (E), and 8 Gy (F). (G, I, K) Representative scatter plots from Annexin V/PI dual‐staining flow cytometry analysis, assessing apoptosis in HIEC‐6 cells from various treatment groups after 0 Gy (G), 4 Gy (I), and 8 Gy (K) irradiation. The “Com” group represents combined treatment with β‐elemene and lactate. (H, J, L) Quantification of the apoptotic rate for each group at irradiation doses of 0 Gy (H), 4 Gy (J), and 8 Gy (L). All data are presented as mean ± SD. Statistical significance was assessed by appropriate statistical tests. *** *p* < 0.001.

We first tested the hypothesis that lactate is the direct protective agent. However, supplementing irradiated cells with a wide range of lactate concentrations (0.5–20 mM) provided no survival benefit. In fact, at higher concentrations (10–20 mM), lactate exhibited modest growth‐inhibitory effects on its own (Figure 4A–C). This finding suggested that lactate is not sufficient and that another factor is required. Based on our in vivo results, we hypothesized that β‐elemene was this missing factor. Indeed, while neither agent alone was effective, co‐administering β‐elemene and lactate resulted in a robust, dose‐dependent rescue of cell viability. An optimal synergy was observed at 100 µg/mL β‐elemene and 5 mM lactate (Figure 4D–F). Mechanistically, this protection stemmed from a potent suppression of radiation‐induced apoptosis (Figure 4G–L).

To determine whether this synergistic effect was mediated by bacterial metabolites, lactate, we compared the effects of *L. gasseri* supernatant and β‐elemene when applied individually or in combination. The results perfectly mirrored our findings with purified lactate: metabolites from *L. gasseri* alone were inert, but in the presence of β‐elemene, they became powerfully protective, restoring cell viability and preventing apoptosis (Figure S4A–G).

Together, these data functionally deconstruct the drug‐microbe‐metabolite axis, revealing a synergistic interplay at the cellular interface. Lactate is the necessary effector molecule, but its protective activity is entirely dependent on a cellular state induced by β‐elemene. This finding raised the central question of how β‐elemene unlocks the protective potential of lactate.

### 3.5 β‐elemene Unlocks Lactate's Protective Potential by Promoting CD147‐Dependent MCT1 Trafficking

**FIGURE 5:**
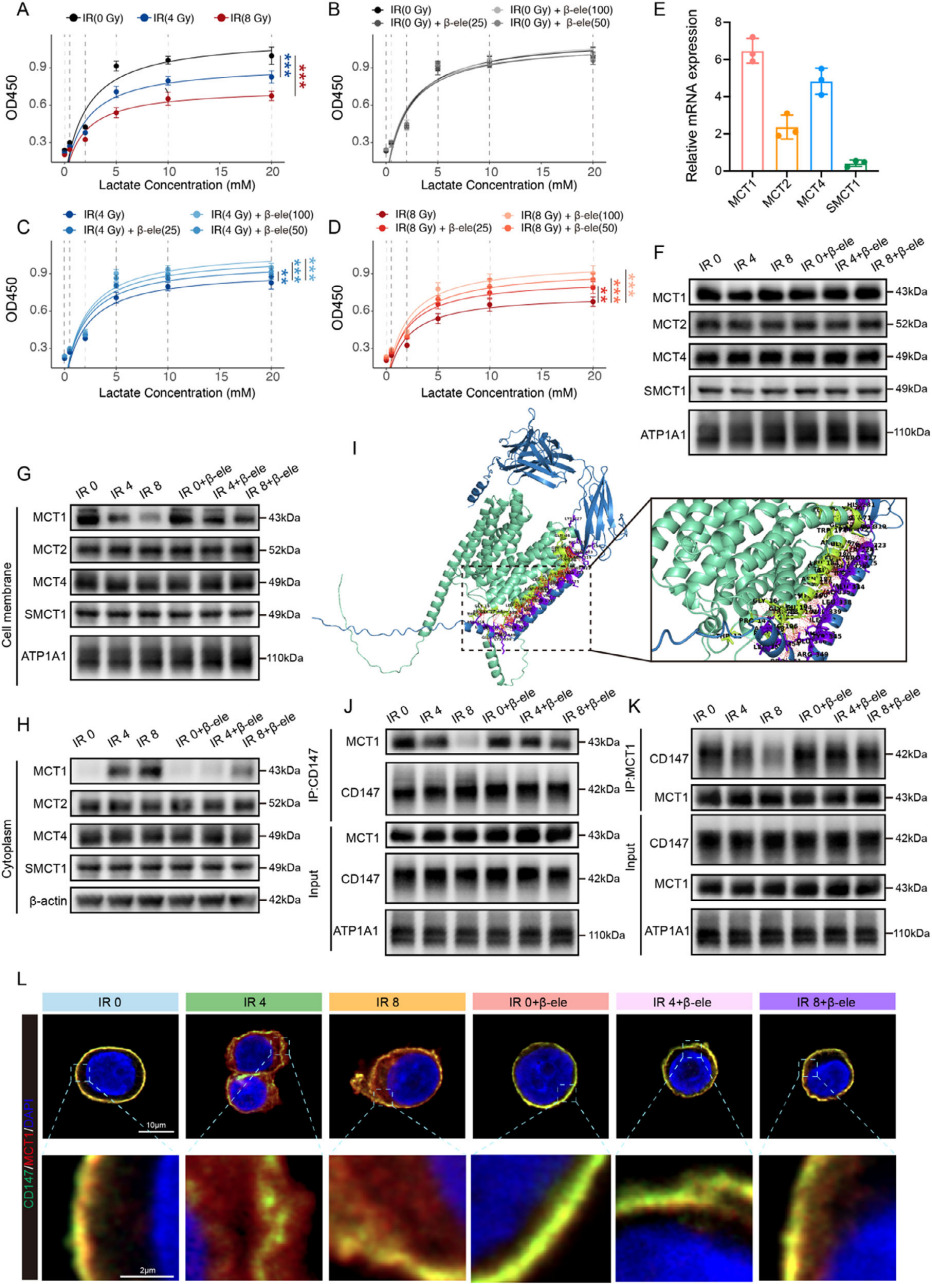
β‐elemene Unlocks Lactate's Protective Potential by Promoting CD147‐dependent MCT1 Trafficking (A–D) Under different doses of IR and β‐elemene pretreatment, the relative intracellular lactate content (represented by OD450 values) was measured by a plate reader after incubating HIEC‐6 cells in media with varying lactate concentrations. The results show that IR inhibited cellular lactate uptake, while β‐elemene could restore this uptake in a dose‐dependent manner. (A) The effect of IR (0, 4, 8 Gy) on lactate uptake. (B) The effect of different β‐elemene concentrations on lactate uptake at 0 Gy. (C) The effect of different β‐elemene concentrations on lactate uptake at 4 Gy. (D) The effect of different β‐elemene concentrations on lactate uptake at 8 Gy. (E) Relative mRNA expression levels of the major monocarboxylate transporters (*MCT1*, *MCT2*, *MCT4*) and the sodium‐coupled monocarboxylate transporter (*SMCT1*) in HIEC‐6 cells, as determined by RT‐qPCR. (F) Western blot analysis of the total protein levels of various transporters in the cell protein fraction from each treatment group (IR: irradiation; β‐ele: β‐elemene). ATP1A1 serves as a cell membrane loading control. (G,H) Western blot analysis of the expression levels of various transporters in cell membrane and cytoplasmic protein fractions. ATP1A1 serves as a cell membrane loading control, and β‐actin as a cytoplasmic loading control. (I) AlphaFold2‐predicted structural model of the interaction between MCT1 (cyan) and its molecular chaperone CD147 (blue). The magnified view on the right shows key interacting residues. (J,K) Co‐immunoprecipitation (Co‐IP) experiments targeting endogenous CD147 (J) and MCT1 (K). (L) Immunofluorescence staining showing the co‐localization (yellow areas) of MCT1 (red) and CD147 (green) on the HIEC‐6 cell membrane. Nuclei were counterstained with DAPI (blue). Scale bar = 10 µm. Original full and unaltered blots can be found in Figure S8.

Our previous results indicated that β‐elemene sensitizes cells to lactate's protective effects. This led us to hypothesize that β‐elemene may function by enhancing cellular lactate uptake. To test this, we measured intracellular lactate levels and found that while irradiation (IR) significantly decreased lactate concentrations in HIEC‐6 cells, β‐elemene treatment robustly reversed this depletion. Notably, β‐elemene had no effect on lactate levels in non‐irradiated cells, suggesting its action is specific to the stress context (Figure 5A–D).

Lactate influx is mediated by monocarboxylate transporters (MCTs) [28, 29]. An expression screen identified monocarboxylate transporter 1 (MCT1) as the most abundant lactate transporter in HIEC‐6 cells (Figure 5E). However, β‐elemene did not alter the total protein levels of MCT1 or other transporters, pointing toward a post‐translational regulatory mechanism, such as altered subcellular localization (Figure 5F). We tested this new hypothesis via cell fractionation and found that β‐elemene treatment significantly increased MCT1 abundance in the membrane fraction, with a corresponding decrease in the cytoplasm. Other transporters were unaffected (Figure 5G,H). This confirms that β‐elemene specifically promotes MCT1 translocation to the plasma membrane.

The trafficking of MCT1 to the cell surface is dependent on its chaperone, cluster of differentiation 147 (CD147) [30]. After confirming their potential interaction with AlphaFold2 (Figure 5I), we used co‐immunoprecipitation (Co‐IP) to validate a physical association between endogenous and exogenous MCT1 and CD147. Importantly, this interaction was significantly weakened by IR, but was fully restored upon β‐elemene treatment (Figure 5J,K; Figure S5A,B). Immunofluorescence analysis corroborated these findings, showing that β‐elemene rescued the co‐localization of MCT1 and CD147 at the cell membrane, which was otherwise disrupted by IR (Figure 5L).

To further investigate whether the restorative effect of β‐elemene on lactate metabolism strictly depends on CD147, we performed loss‐of‐function experiments using siRNA targeting CD147 (si‐CD147). Membrane fractionation combined with Western blot analysis revealed that CD147 knockdown completely abolished the β‐elemene‐induced translocation of MCT1 to the cell membrane following irradiation, trapping MCT1 in the cytoplasm (Figure S5C). Consistently, subsequent functional assays demonstrated that while β‐elemene significantly promoted lactate uptake in irradiated cells, this metabolic rescue effect was entirely abrogated in CD147‐knockdown cells (Figure S4D). Collectively, these data indicate that β‐elemene facilitates intracellular lactate uptake and exerts its protective effects in a precisely CD147‐dependent manner, relying on the stabilization of the MCT1‐CD147 complex.

### 3.6 The β‐elemene‐Lactate Synergy Drives a POLD1/POLD3‐Dependent DNA Damage Repair Program

**FIGURE 6:**
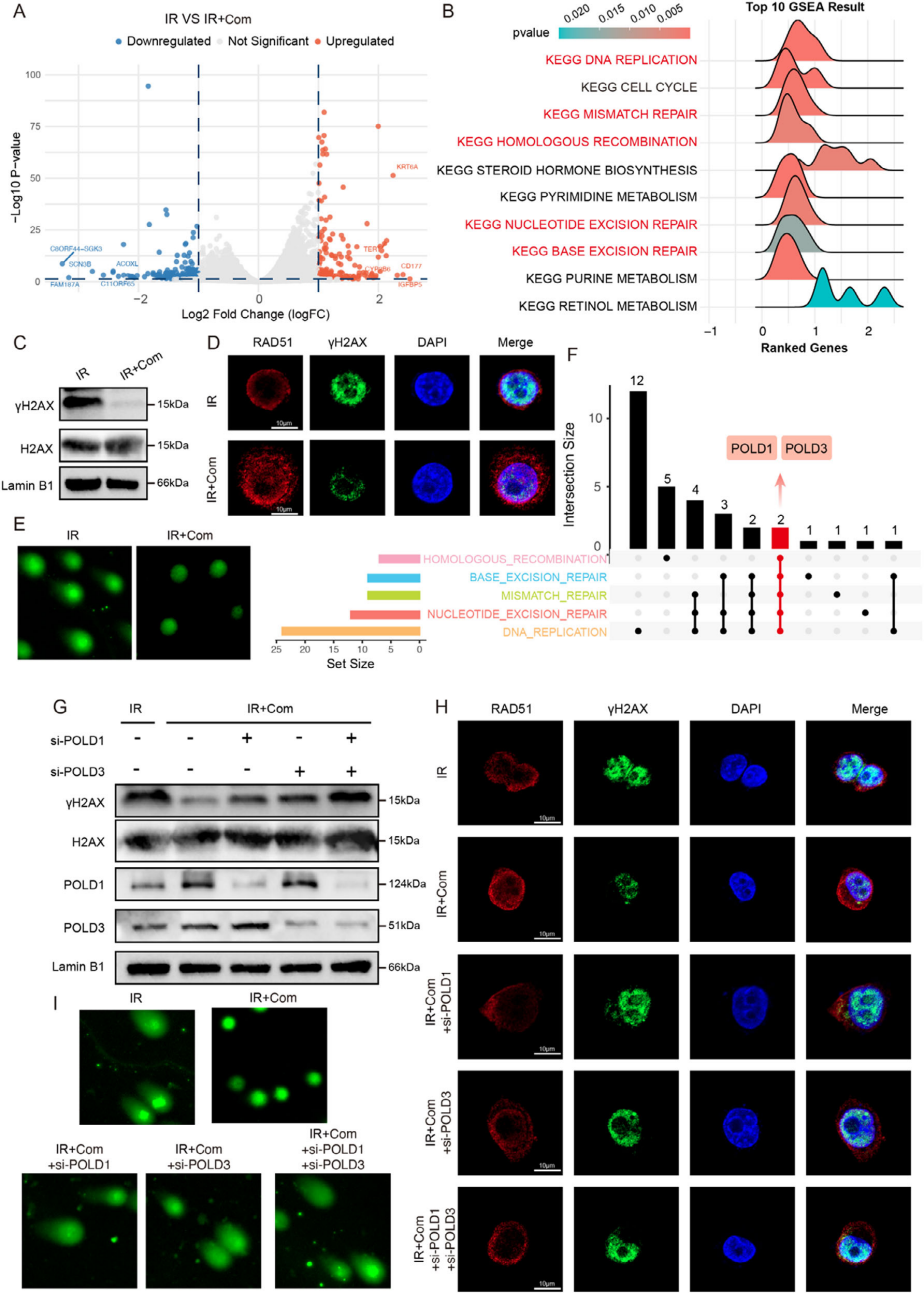
The β‐elemene‐lactate Synergy Drives a POLD1/POLD3‐dependent DNA Damage Repair Program (A) Volcano plot comparing RNA sequencing results from HIEC‐6 cells in the abdominal irradiation (IR) group and the combination treatment (IR+Com) group, with the most significant differentially expressed genes labeled. (B) GSEA results showing significant enrichment of KEGG pathways related to DNA replication and multiple DNA damage repair mechanisms in the combination treatment group. (C) Western blot analysis of the protein levels of the double‐strand break marker γH2AX in the IR and IR+Com groups. Lamin B1 serves as a nuclear loading control. (D) Immunofluorescence staining showing the expression and localization of the key homologous recombination protein RAD51 (red) and γH2AX (green) within the nucleus. Nuclei were counterstained with DAPI (blue). (Scale bar = 10 µm). (E) Images from a Comet assay used to assess the degree of cellular DNA damage. A longer “comet tail” represents more severe DNA damage. (F) UpSet plot displaying genes shared among the enriched DNA repair pathways. *POLD1* and *POLD3* are identified as core genes common to multiple key pathways. (G) Western blot results after knocking down *POLD1* and *POLD3*, respectively, using siRNA in IR+Com‐treated cells. The results show that knocking down *POLD1* or *POLD3* reverses the inhibitory effect of the combination treatment on γH2AX. (H) Immunofluorescence images corresponding to the gene knockdown experiment in (G). Knockdown of *POLD1* or *POLD3* attenuated the combination treatment‐induced formation of RAD51 foci and increased the accumulation of γH2AX. (Scale bar = 10 µm). (I) Comet assay images corresponding to the gene knockdown experiment in (G). The results show that knocking down *POLD1* or *POLD3* abrogates the DNA damage repair effect of the combination treatment. Original full and unaltered blots can be found in Figure S9.

To elucidate the downstream molecular mechanisms of the synergistic protection, we performed an unbiased transcriptomic analysis (RNA‐seq) on irradiated HIEC‐6 cells with or without combination treatment (Figure 6A). Gene Set Enrichment Analysis (GSEA) revealed that the gene expression profile of the combination treatment group was significantly enriched for pathways related to DNA Replication and DNA Damage Repair (Figure 6B, Table S8), consistent with the observed pro‐survival phenotype.

We then sought to functionally validate this pathway‐level finding. Combination treatment with β‐elemene and lactate significantly reduced the levels of γH2AX, a specific marker for DNA double‐strand breaks (DSBs) [31, 32], as confirmed by both Western blot and immunofluorescence (Figure 6C,D). To directly assess repair kinetics, we performed a neutral comet assay. The results showed that combination treatment cells exhibited a significantly shorter comet tail moment 6 h post‐irradiation, indicating more efficient repair of DSBs (Figure 6E).

To identify the key effector genes responsible for this enhanced repair capacity, an intersectional analysis of differentially expressed genes (DEGs) using an UpSet plot identified *POLD1* and *POLD3* (Figure 6F). Both genes encode essential subunits of DNA Polymerase delta (Pol‐δ), a critical enzyme in DNA replication and repair [33, 34, 35]. To establish their necessity, we performed siRNA‐mediated knockdown of each gene. Depletion of either *POLD1* or *POLD3* was sufficient to largely abrogate the protective effects of the combination treatment, leading to increased γH2AX levels and impaired DNA repair in the comet assay (Figure 6G–I; Figure S6A,B). Supporting this mechanistic link, analysis of the GEPIA2 database showed a significant positive correlation between the *MCT1* and both *POLD1* (R = 0.35, p < 0.05) and *POLD3* (R = 0.23, p < 0.05) in normal gastrointestinal tissues (Figure S6C,D).

Collectively, these results demonstrate that the synergy between lactate and β‐elemene protects HIEC‐6 cells by transcriptionally upregulating *POLD1* and *POLD3*, thereby enhancing Pol‐δ‐mediated DNA repair and promoting genomic integrity after radiation exposure.

### 3.7 The β‐elemene‐Lactate Synergy Drives POLD1/POLD3 Transcription via an EP300‐Mediated H3K27ac Switch

**FIGURE 7:**
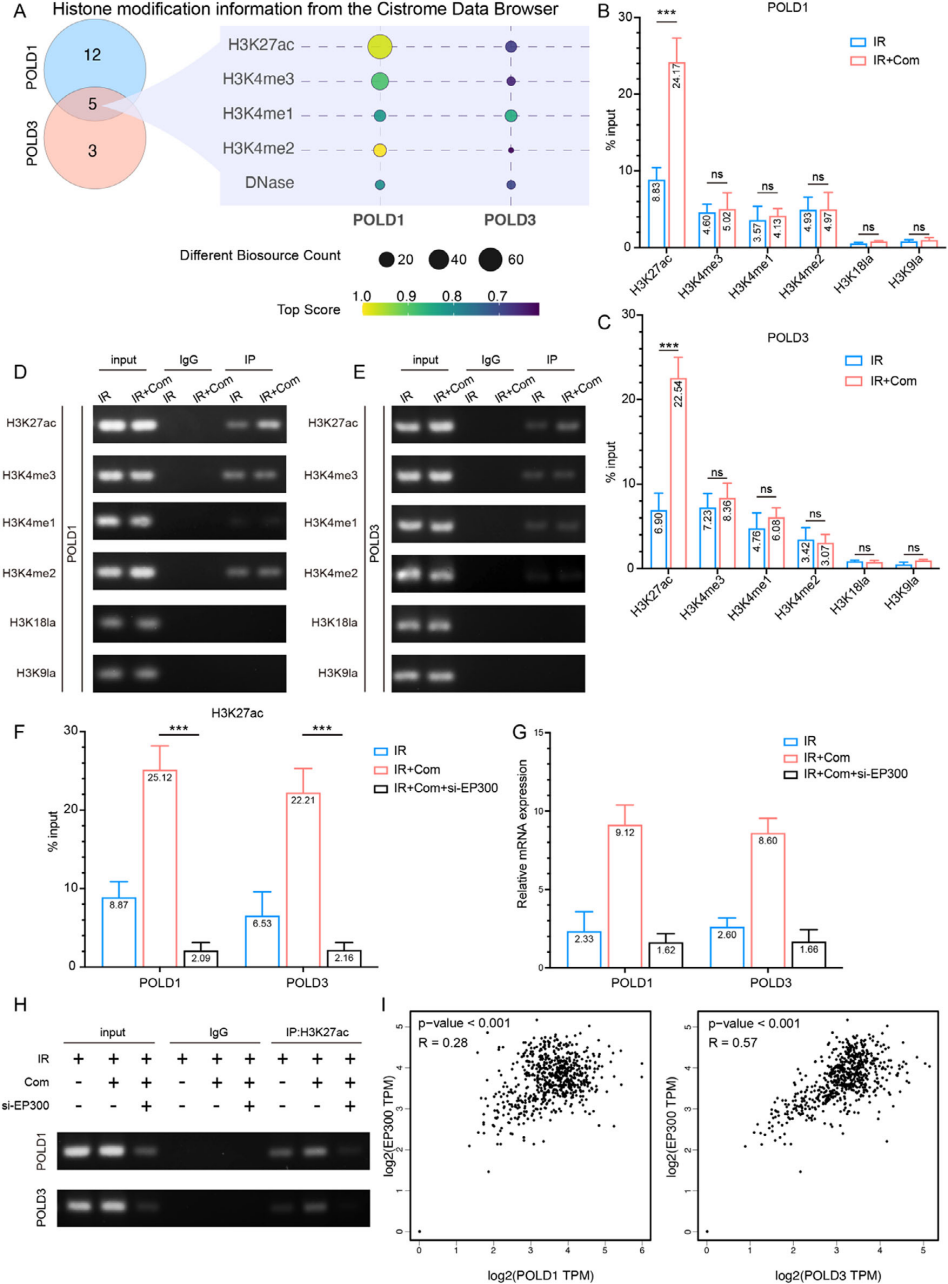
The β‐elemene‐lactate Synergy Drives *POLD1*/*POLD3* Transcription via an EP300‐mediated H3K27ac Switch (A) Analysis based on the Cistrome Data Browser. The Venn diagram on the left shows the overlap of histone modifications associated with the *POLD1* and *POLD3* genes. The bubble plot on the right displays the enrichment scores for various active histone marks (H3K27ac, H3K4me3, etc.) on the *POLD1* and *POLD3* genes. (B,) ChIP‐qPCR analysis of the promoter regions of *POLD1* (B) and *POLD3* (C). (D,E) Corresponding semi‐quantitative ChIP‐PCR gel electrophoresis images for (B) and (C), visually demonstrating the enrichment of H3K27ac in the promoter regions of *POLD1* (D) and *POLD3* (E) in the combination treatment group. (F) ChIP‐qPCR analysis of H3K27ac levels at the POLD1 and POLD3 promoter regions after knockdown of the histone acetyltransferase EP300 (si‐EP300). (G) Relative mRNA expression levels of *POLD1* and *POLD3* after EP300 knockdown, as determined by RT‐qPCR. (H) Semi‐quantitative ChIP‐PCR gel electrophoresis image corresponding to (F). (I) Gene expression correlation analysis based on the GEPIA2 database. The scatter plots show that the expression level (TPM) of EP300 is significantly positively correlated with the expression levels of both *POLD1* (left, R = 0.28) and *POLD3* (right, R = 0.57) (*p* < 0.001). All quantitative data are presented as mean ± SD. ns, not significant; *** *p* < 0.001.

Having identified *POLD1* and *POLD3* as key downstream genes, we next investigated their upstream transcriptional regulation. An initial bioinformatic screen using the Cistrome Data Browser indicated that the promoters of both genes are enriched with several histone marks associated with active transcription, prominently including H3K27ac, H3K4me3, H3K4me1, and H3K4me2 (Figure 7A).

To determine which of these marks were dynamically regulated by our combination treatment, we performed chromatin immunoprecipitation with quantitative PCR (ChIP‐qPCR). We assayed for these four activating marks, and in light of the elevated intracellular lactate, we also included two histone lactylation marks (H3K18la, H3K9la). Of the six modifications tested, only H3K27ac showed a significant and consistent enrichment at both the *POLD1* and *POLD3* promoters following combination treatment. This finding pinpointed H3K27ac as the key epigenetic event driving the transcriptional response (Figure 7B–E).

The histone acetyltransferase (HAT) EP300 is a primary “writer” of H3K27ac [36, 37]. To test its causal role, we depleted EP300 using siRNA. The knockdown of EP300 was sufficient to prevent the combination treatment‐induced enrichment of H3K27ac at the *POLD1* and *POLD3* promoters (Figure 7F,H). Critically, this loss of the epigenetic mark directly translated to a functional consequence: the transcriptional upregulation of *POLD1* and *POLD3* mRNA was also significantly attenuated (Figure 7G). Consistent with this regulatory axis, analysis of the GEPIA2 database showed significant positive correlations between *EP300* expression and that of *POLD1* (R = 0.28, p < 0.05) and *POLD3* (R = 0.57, p < 0.05) in normal gastrointestinal tissues (Figure 7I).

Thus, these data delineate a clear epigenetic pathway where the synergy between β‐elemene and lactate promotes EP300‐dependent H3K27 acetylation at the *POLD1* and *POLD3* promoters to drive their transcription. This, however, raised the critical question of how elevated intracellular lactate directs EP300 activity to these specific genomic loci.

### 3.8 Lactylation of RBBP4 at Lysine 26 Is Required for EP300 Recruitment and H3K27ac Deposition at POLD1/POLD3 Promoters

**FIGURE 8:**
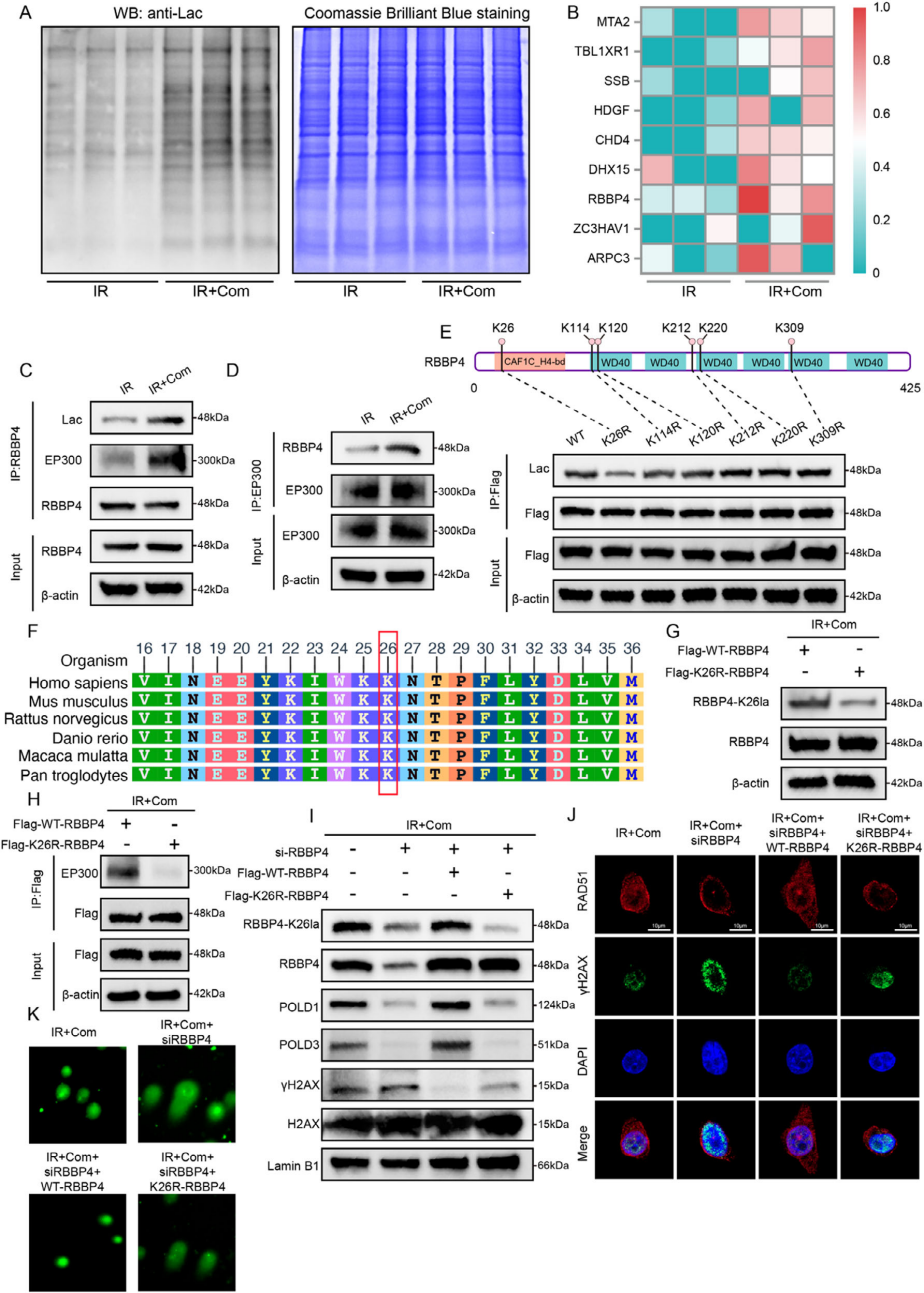
Lactylation of RBBP4 at Lysine 26 Is Required for EP300 Recruitment and H3K27ac Deposition at *POLD1*/*POLD3* Promoters (A) Western blot (WB) analysis showing that combination treatment (IR+Com) significantly increased global protein lactylation (Lac) levels within cells. The panel on the right is a Coomassie brilliant blue stain serving as a loading control. (B) Heatmap of proteomics (mass spectrometry) analysis after pulldown with a pan‐lactylation antibody, displaying proteins with significantly upregulated lactylation levels in the combination treatment group. (C‐D) Co‐immunoprecipitation (Co‐IP) experiments. IP was performed using an anti‐RBBP4 antibody (C) and an anti‐EP300 antibody (D), respectively. (E) Identification of the key lactylation site on RBBP4. Top: Schematic diagram of RBBP4 protein domains. Bottom: WB results showing the detection of lactylation signals in various lysine (K) to arginine (R) mutants. (F) Sequence alignment of the RBBP4 protein across different species, showing that the K26 site (indicated by the red box) is highly conserved. (G,H) Co‐IP experiment after overexpressing Flag‐tagged wild‐type (WT) or K26R mutant RBBP4, demonstrating that the K26 mutation impairs the interaction between RBBP4 and EP300. (I) WB analysis of an RBBP4 knockdown and rescue experiment. After knocking down endogenous RBBP4, re‐expression of WT‐RBBP4 restored POLD1/POLD3 expression and inhibited γH2AX levels, whereas the K26R mutant did not. Lamin B1 serves as a nuclear loading control. (J) Immunofluorescence staining showing the expression and localization of the key homologous recombination protein RAD51 (red) and γH2AX (green) within the nucleus. Nuclei were counterstained with DAPI (blue). (Scale bar = 10 µm). (K) Comet assay images corresponding to the gene knockdown and rescue experiment in (I). Original full and unaltered blots can be found in Figure S10.

We had established that the β‐elemene‐lactate synergy requires EP300‐dependent H3K27ac to upregulate *POLD1*/*POLD3*. However, the mechanism linking elevated intracellular lactate to the targeted recruitment of EP300 remained unknown. We hypothesized that a lactylated protein might serve as a molecular bridge. Indeed, combination treatment increased global protein lactylation in HIEC‐6 cells (Figure 8A).

To identify this bridging protein, we employed a dual‐screening strategy. Employing anti‐L‐lactyl lysine (Kla) antibody‐based IP coupled with 4D‐FastDIA proteomics, we identified nine proteins that showed significantly increased lactylation upon combination treatment (Figure 8B, Table S9). Concurrently, a search of the GTRD database predicted potential transcription regulators binding to the *POLD1* and *POLD3* promoters. Intersecting these two datasets narrowed the candidates to retinoblastoma‐binding protein 4 (RBBP4) and Transducin Beta Like 1 X‐Linked Receptor 1 (TBL1XR1) (Figure S7A). Subsequent ChIP‐qPCR validation confirmed that only RBBP4 specifically binds to the *POLD1* and *POLD3* promoter regions (Figure S7B,C). Given that RBBP4 is a known interactor of EP300, we focused our investigation on this protein.

We first confirmed that endogenous RBBP4 is indeed lactylated and that this modification is significantly enhanced by combination treatment (Figure 8C). This enhanced lactylation correlated with a stronger physical interaction between RBBP4 and EP300 (Figure 8D). To pinpoint the specific modification site, we used DeepKla to predict six conserved lysine residues (K26, K114, K120, K212, K220, K309) and systematically mutated each to arginine (K‐to‐R) [38]. Only the K26R mutant completely lost its ability to be lactylated in response to combination treatment (Figure 8E), a site that is highly conserved across species (Figure 8F).

To directly probe the function of this specific modification, we developed a custom antibody that specifically recognizes lactylated RBBP4 at K26 (RBBP4‐K26la). This antibody confirmed that endogenous RBBP4‐K26la is induced by combination treatment and is specific to the wild‐type (WT), not the K26R mutant (Figure 8G). Crucially, Co‐IP experiments revealed that the K26R mutation significantly weakened the interaction between RBBP4 and EP300 (Figure 8H).

To definitively establish the causal role of K26 lactylation in the entire signaling cascade, we performed a knockdown‐rescue experiment. While re‐expression of WT RBBP4 in RBBP4‐depleted cells restored the entire protective program, the non‐lactylatable K26R mutant failed to do so. Specifically, the K26R mutant was unable to rescue combination treatment‐induced H3K27ac enrichment at the *POLD1*/*POLD3* promoters, could not restore *POLD1*/*POLD3* expression, and failed to reduce the DNA damage marker γH2AX or improve cell survival phenotypes (Figure 8I–K; Figure S7D–G). This functional link is further supported by significant correlations between *RBBP4* and *EP300*, *POLD1*, and *POLD3* in the GEPIA2 database (Figure S7H).

In conclusion, these results demonstrate that lactylation of RBBP4 at K26 is the essential molecular event that strengthens its interaction with EP300, thereby recruiting the acetyltransferase to the *POLD1* and *POLD3* promoters to drive a protective DNA repair program.

### 3.9 Functions as the Lactyl‐Transferase for RBBP4 to Establish a Positive Feedback Loop

**FIGURE 9:**
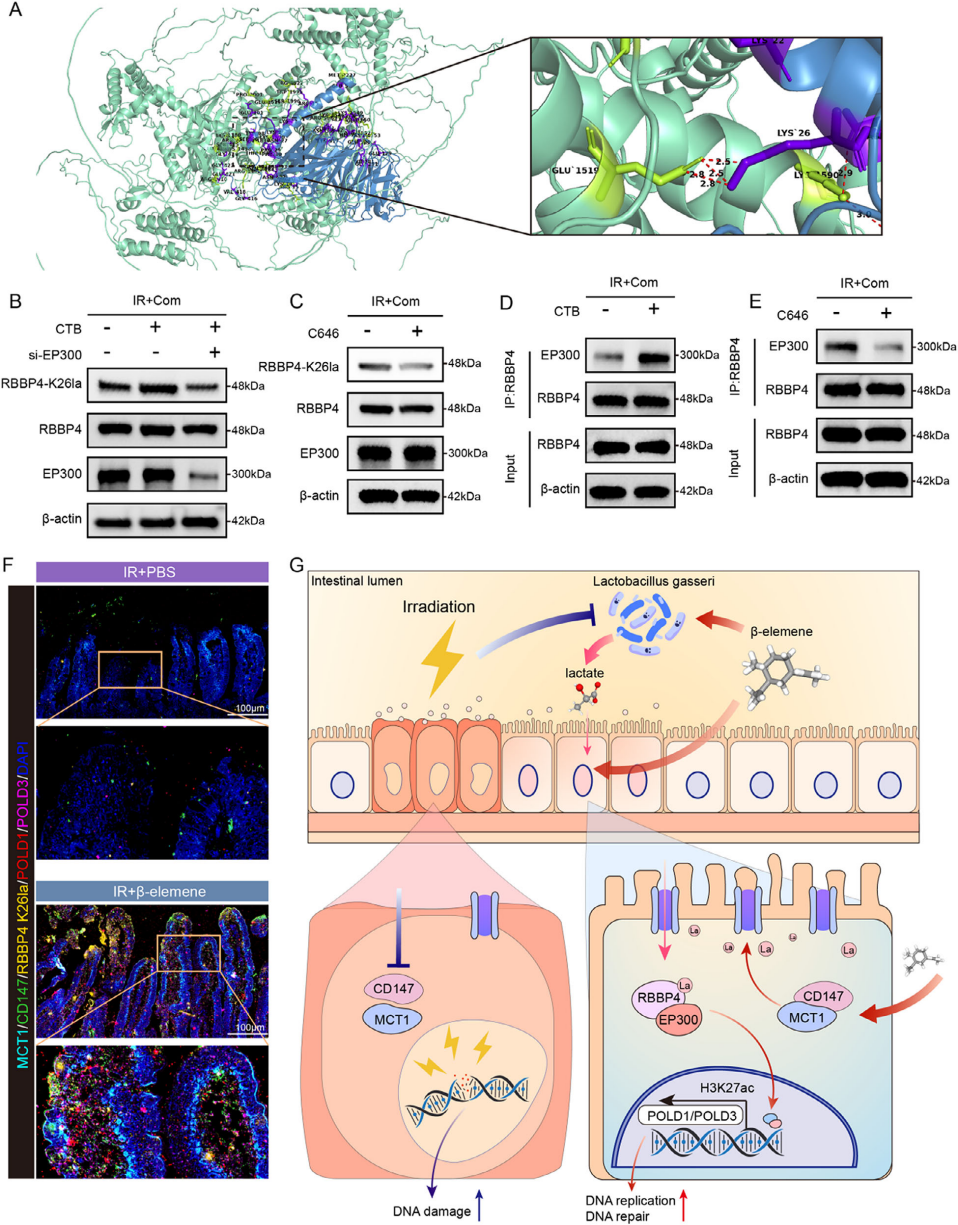
EP300 Functions as the Lactyl‐transferase for RBBP4 to Establish a Positive Feedback Loop (A) Molecular docking model. Left: Overall predicted structure of the interaction between a lactylated RBBP4 peptide (light blue) and the EP300 protein (cyan). Right: Magnified view showing that the K26 lactylation (Lys 26) on RBBP4 can form hydrogen bonds with key residues such as Glutamic Acid (GLU 1519) in the EP300 catalytic domain, thereby stabilizing the interaction. (B,C) Western blot (WB) analysis. (D,E) Co‐immunoprecipitation (Co‐IP) experiments with IP performed using an anti‐RBBP4 antibody (D) and an anti‐EP300 antibody (E). The results indicate that The EP300 agonist CTB enhanced (D), while the inhibitor C646 reduced (E), the interaction between EP300 and RBBP4. (F) Multiplex immunofluorescence staining of mouse ileal tissues. Compared to the radiation‐only group (IR+PBS), the β‐elemene treatment group (IR+β‐elemene) showed significantly enhanced expression and co‐localization of MCT1 (cyan), CD147 (green), RBBP4‐K26la (yellow), POLD1 (red), and POLD3 (purple) in the intestinal crypt regions. (G) Summary and schematic model of the mechanism. This study elucidates that β‐elemene promotes cellular uptake of *L. gasseri*‐derived lactate by enhancing the membrane translocation of MCT1 in a CD147‐dependent manner. Intracellular lactate, through the lactylation of RBBP4 at the K26 site, recruits EP300 to the promoter regions of downstream genes (*POLD1*/*POLD3*), catalyzing H3K27ac modification. This ultimately activates the DNA damage repair pathway, protecting intestinal epithelial cells from radiation injury. Original full and unaltered blots can be found in Figure S11.

Our finding that lactylated RBBP4 recruits EP300 led to a final critical question: which enzyme is the “writer” for the RBBP4 K26 lactylation? As EP300 itself is known to possess lactyl‐transferase activity [39, 40, 41], we explored the structural basis for this potential interaction using AlphaFold2. The protein docking model predicted that the K26 residue of RBBP4 lies directly within the interaction interface of EP300's catalytic domain (Figure 9A). This suggested the possibility of a positive feedback loop, where EP300 directly lactylates its own recruitment factor, RBBP4, to amplify the signaling cascade.

To functionally test this hypothesis, we employed pharmacological and genetic tools. First, we demonstrated that the EP300‐specific agonist CTB robustly induced RBBP4 lactylation. Crucially, this induction was completely abrogated in cells depleted of EP300 by siRNA, establishing that EP300 is the indispensable mediator of this modification (Figure 9B). Conversely, the selective EP300 inhibitor C646 significantly reduced basal RBBP4 lactylation levels (Figure 9C). These catalytic effects directly correlated with the physical interaction, as Co‐IP experiments showed that CTB enhanced, while C646 inhibited, the association between EP300 and RBBP4 (Figure 9D,E).

Finally, we sought to validate this entire mechanistic axis in vivo. Multiplex immunohistochemistry (mIHC) on intestinal tissues from our animal model revealed the precise molecular signatures predicted by our findings. Compared to the irradiation group, tissues from mice treated with β‐elemene exhibited increased MCT1 membrane localization, elevated levels of RBBP4 K26 lactylation, and a significant upregulation of *POLD1* and *POLD3* (Figure 9F). Collectively, these data close the loop on our investigation, demonstrating that EP300 acts as the direct lactyl‐transferase for RBBP4, creating a self‐reinforcing mechanism that translates a microbe‐derived metabolic signal into a robust, radioprotective DNA repair program.

## 4 Discussion

This study elucidates a novel, multi‐kingdom mechanism of action for β‐elemene in protecting against radiation‐induced enteritis, significantly advancing our understanding of how this natural product exerts its therapeutic effects. The central discovery is that β‐elemene orchestrates a sophisticated, two‐pronged synergistic strategy that bridges pharmacology, microbiology, and host epigenetics. It operates through a dual mechanism: concurrently remodeling the gut microbiome to increase the supply of a protective metabolite and directly priming host intestinal epithelial cells to effectively utilize that same metabolite (Figure 9G). This ‘prime‐and‐fuel’ model, dissected from the organismal to the atomic level in this study, represents a significant conceptual advance, revealing a molecular playbook by which a single agent can coordinate inter‐kingdom communication to promote tissue homeostasis and repair.

Our investigation began by validating the central hypothesis that β‐elemene's efficacy is critically dependent on the gut microbiota. The complete abrogation of its protective effects against radiation‐induced weight loss, intestinal shortening, and severe histological damage in antibiotic‐treated mice unequivocally demonstrated that the microbiota is an indispensable mediator of its action [42, 43]. We further dissected this dependency and found that β‐elemene acts with remarkable specificity, selectively enriching for the commensal bacterium *L. gasseri*, a well‐characterized probiotic [44, 45]. This targeted modulation had a direct functional consequence: a significant elevation in intestinal lactate levels. The causal link was definitively established in germ‐free mice, where radioprotection was restored only upon the co‐administration of *L. gasseri* and β‐elemene, confirming that the drug drives the production of a key, microbe‐derived therapeutic effector, which has been increasingly recognized as a critical signaling molecule in wound healing and immune regulation [46, 47, 48, 49].

A striking and pivotal observation in our study is the specificity with which β‐elemene enriches *L. gasseri* in vivo. While our data conclusively link this enrichment to downstream radioprotection, the exact mechanism driving this microbial selectivity warrants further investigation. While the exact ecological trigger remains to be fully elucidated, we hypothesize that β‐elemene may shape this specific microbial niche either through direct selective antimicrobial effects against radiation‐induced pathobionts or by preserving epithelial hypoxia to favor *L. gasseri* [50, 51, 52]. Future studies focusing on bacterial growth kinetics in vitro and multi‐omics analysis of mucosal biofilms will be instrumental in fully deciphering this initial trigger of the prime‐and‐fuel cascade. To definitively elucidate the specific weight of these respective pathways, our ongoing and future research will employ in vitro multi‐species co‐culture models to assess direct bacterial competition, combined with dynamic spatial transcriptomics and mucosal metabolomics in vivo to precisely map the early temporal events of this microbe‐drug interplay and the restructuring of the host niche.

Our study was initially guided by the hypothesis that β‐elemene's protective effects were mediated entirely through the gut microbiota. While the abrogation of its efficacy in microbiota‐depleted mice strongly supported this premise, our subsequent findings revealed the more intricate ‘prime‐and‐fuel’ mechanism. We discovered that β‐elemene exerts a dual action: it directly primes host intestinal epithelial cells by stabilizing the MCT1‐CD147 complex, thereby enhancing their capacity for lactate uptake, while simultaneously remodeling the microbiome to increase lactate availability. This refined model, where the drug coordinates both host and microbial responses, represents a significant conceptual advance over a purely microbe‐centric mechanism.

This discovery raised a more nuanced question: how do host cells, particularly in a radiation‐damaged environment, perceive and leverage the lactate signal provided by the microbiota? Our investigation revealed that the answer lies in a previously unknown facet of β‐elemene's mechanism, positioning it as a critical modulator of host‐microbe metabolic crosstalk. Our in vitro analyses revealed a striking pharmacological synergy. When administered alone, lactate, even across a range of physiological concentrations, failed to rescue irradiated intestinal epithelial cells from apoptosis. This crucial negative result suggests that under the pathological stress of irradiation, the host cell machinery for utilizing lactate is fundamentally impaired, creating a metabolic bottleneck. However, the introduction of β‐elemene completely reversed this paradigm. In its presence, lactate triggered a robust, dose‐dependent rescue of cell viability and a potent suppression of apoptosis. This led us to the discovery that β‐elemene acts directly on host cells as a “patho‐regulator,” priming them to effectively utilize beneficial microbial metabolites. Delving into the underlying mechanism, we provide converging lines of evidence from cell fractionation, co‐immunoprecipitation, and immunofluorescence that this bottleneck is structural. We demonstrate that ionizing radiation causes a functional uncoupling of the lactate transporter MCT1 from its essential chaperone protein, CD147. This disruption is a critical pathogenic event, as the MCT1‐CD147 interaction is indispensable for MCT1's stability and translocation to the plasma membrane [30, 53, 54]. Our data demonstrate that β‐elemene effectively preserves this interaction under radiation stress, potentially acting as a molecular stabilizer or “pharmacological chaperone.” By preserving the integrity of the MCT1‐CD147 complex, β‐elemene promotes MCT1's proper trafficking to the cell surface, thereby restoring the cell's capacity to import lactate. This direct priming of host cell machinery is the crucial event that unlocks lactate's latent therapeutic potential, transforming it from an inert metabolite into a potent radioprotective agent. Importantly, our data reveal that β‐elemene does not affect MCT1 membrane localization or intracellular lactate uptake in normal, non‐irradiated cells. This finding indicates that β‐elemene functions fundamentally as a “pharmacological chaperone” rather than a constitutive transport activator. In unstressed cells, where the native MCT1‐CD147 complex is structurally intact and functionally stable, β‐elemene does not perturb baseline metabolic homeostasis. Its restorative efficacy is exclusively unlocked under pathological conditions, specifically when the complex is physically uncoupled by genotoxic stress. This highly context‐dependent action provides a compelling mechanistic explanation for the wide therapeutic window and excellent safety profile of β‐elemene observed in clinical practice [55]. By functioning only when the system is perturbed, it precisely rescues damaged tissues without inadvertently forcing aberrant metabolic reprogramming in healthy host cells.

The observation that IR inhibits the functional binding of MCT1 and CD147 opens a critical avenue for understanding radiation pathology. While our study establishes this disruptive event and identifies β‐elemene as a counteracting stabilizer, the precise upstream molecular trigger initiated by ionizing radiation warrants thorough investigation. We hypothesize that this uncoupling is primarily driven by a “dual‐hit” mechanism induced by severe oxidative stress. First, from a structural perspective, the chaperone function of CD147 heavily relies on highly conserved disulfide bonds within its extracellular Ig‐like domains [54, 56]. The acute burst of reactive oxygen species (ROS) following radiation exposure likely induces aberrant oxidation of these critical cysteine residues, resulting in a conformational shift that sterically dismantles the MCT1‐CD147 interaction interface [57]. Alternatively, IR may trigger stress‐responsive kinases (e.g., ATM/ATR) leading to inhibitory phosphorylation events that hinder complex formation [58]. Second, from a spatial perspective, IR‐mediated lipid peroxidation perturbs the integrity of lipid raft microdomains, which are essential for anchoring this transport complex at the plasma membrane, thereby causing their physical segregation [59]. Correspondingly, the mechanisms by which the lipophilic sesquiterpene β‐elemene counteracts this disruption likely operate precisely at this interface. It may partition into the lipid bilayer acting as a ‘pharmacological chaperone’ to directly stabilize the transmembrane assembly against structural perturbations, or serve as a localized antioxidant to preserve lipid raft integrity [60, 61]. Future redox‐proteomic studies and phosphoproteomic profiling will be crucial to map these specific post‐translational modifications and fully validate this upstream signaling cascade. Crucially, the mechanistic paradigm delineated by our in vitro Co‐IP and cellular assays was faithfully recapitulated in vivo. As explicitly demonstrated by our mIHC analysis of the intestinal tissues (Figure 9F), β‐elemene treatment robustly restored the spatial colocalization of MCT1 and CD147 within the crypt epithelium of irradiated mice. This provides definitive in situ validation of the transporter‐rescue mechanism in the actual physiological niche responsible for tissue regeneration, seamlessly bridging our molecular findings with the organismal radioprotective phenotype.

A critical point of discussion is the context‐dependent duality of β‐elemene's function. While extensively documented as a pro‐apoptotic agent in various cancer models [62], our study reveals a pro‐survival and pro‐reparative role in non‐malignant intestinal epithelial cells subjected to acute genotoxic stress. We hypothesize that this apparent paradox stems from the fundamentally different cellular states and biological objectives of these two cell types. Cancer cells, driven by the Warburg effect, are highly glycolytic and rely critically on MCT1 (often alongside MCT4) primarily for lactate efflux to prevent lethal intracellular acidification and maintain tumor energetics [63, 64, 65, 66, 67, 68, 69]. In this context, any stabilization of MCT1 by β‐elemene might paradoxically force these cells to confront their own toxic metabolic waste if the extracellular lactate concentration is high, or it simply cannot counteract the massive, catastrophic DNA double‐strand breaks directly inflicted by high‐dose tumor‐targeted radiotherapy. In contrast, the primary biological imperative of a normal cell exposed to acute radiation is not aberrant proliferation but damage control and survival [70, 71]. Normal intestinal crypt cells act as crucial metabolic consumers, utilizing MCT1 primarily for the influx of microbiome‐derived metabolites for tissue homeostasis [72, 73]. Therefore, β‐elemene's stabilization of the MCT1‐CD147 complex acts as a facilitator for normal cells, providing the necessary fuel for endogenous repair, while potentially offering no survival advantage—or even adding metabolic stress—to Warburg‐dependent cancer cells. Future investigations utilizing tumor‐bearing mice are imperative to definitively confirm that β‐elemene's ‘prime‐and‐fuel’ radioprotective mechanism does not compromise the therapeutic efficacy of ionizing radiation against abdominal malignancies.

The therapeutic power of β‐elemene thus resides in the elegant convergence of these two parallel actions. It simultaneously acts as a “microbiome conditioner” to increase the local supply of lactate and as a “host‐cell primer” to enhance the machinery for lactate uptake. The integration of these synergistic signals triggers a robust downstream metabo‐epigenetic cascade, which we identified through unbiased transcriptomic analysis. We have delineated this pathway from signal reception to genomic output, showing that the influx of lactate leads to the lactylation of the chromatin‐associated protein RBBP4 at lysine 26. This novel post‐translational modification, identified from a pool of potential histone and non‐histone targets, serves as a molecular beacon that enhances RBBP4's interaction with the acetyltransferase EP300. This targeted recruitment leads to H3K27ac deposition specifically at the promoters of the DNA Polymerase δ subunits *POLD1* and *POLD3*, driving their transcription and enhancing the cell's capacity for DNA repair. Finally, the identification of EP300 as the lactyl‐transferase for RBBP4 establishes a self‐reinforcing positive feedback loop that ensures a stable and potent activation of this pro‐regenerative program. While this EP300‐RBBP4 positive feedback loop provides a potent, self‐amplifying engine for rapid DNA repair, its timely termination is equally critical to restore cellular homeostasis once genomic integrity is re‐established. We postulate that the resolution of this metabo‐epigenetic cascade is orchestrated by multi‐tiered ‘off‐switches’. Initially, a natural metabolic shift likely occurs: as tissue repair concludes, the transient hyper‐influx of microbe‐derived lactate normalizes, thereby depriving EP300 of its acyl‐donor substrate and naturally dampening the feed‐forward cycle [74]. Concurrently, active enzymatic termination may be driven by epigenetic ‘erasers’. Negative regulators, such as classical histone deacetylases (HDACs) or Sirtuins (e.g., SIRT1/2/3) known for their diverse de‐acylase activities, might be recruited during the late stages of repair to specifically remove the K26la mark on RBBP4 and H3K27ac marks on target promoters [75]. Alternatively, post‐repair structural dismantling via ubiquitin‐proteasome degradation of these modified complexes could also contribute to resetting the chromatin landscape [76]. To precisely define these termination dynamics, our ongoing studies will utilize extended time‐course metabolomics paired with specific pharmacological inhibition of candidate deacetylases (e.g., HDAC and SIRT inhibitors) to map the spatiotemporal resolution of this ‘prime‐and‐fuel’ cascade. While our independent validation cohorts provide strong functional evidence for the β‐elemene‐lactate axis, we recognize the current methodological challenge in achieving precise quantitative partitioning of host‐ versus microbiome‐derived lactate molecules in vivo. Definitive tracking of inter‐kingdom metabolic flux via 13C‐isotope tracing in gnotobiotic models remains a technical frontier. Due to current infrastructural constraints for specialized anaerobic isotopic labeling, this high‐resolution metabolic mapping is designated as the primary focus of our future collaborative investigations to fully delineate the atomic‐level carbon flux within this host‐microbe circuit.

From a translational perspective, the clinical feasibility of β‐elemene is highly promising. Based on FDA conversion guidelines [77], our optimal murine dose (50 mg/kg) translates to a Human Equivalent Dose (HED) of ∼243 mg/day for a 60 kg adult, which falls safely below the standard clinical oncology doses of 400–600 mg/day [55]. Furthermore, while standard clinical administration achieves plasma concentrations in the range of 3–12 µg/mL, β‐elemene is a highly lipophilic sesquiterpene [55, 78]. It rapidly partitions from the aqueous plasma into lipid‐rich cellular membranes [79]. This lipophilic accumulation ensures that the local drug concentration at the intestinal epithelial membrane—precisely where the MCT1‐CD147 target complex resides—is sufficiently high to exert its stabilizing chaperone effect, effectively bridging the apparent gap between in vivo plasma levels and our in vitro experimental concentrations (100 µg/mL).

In conclusion, this study redefines β‐elemene as a master orchestrator of a protective host‐microbe circuit. The “prime‐and‐fuel” strategy represents a new therapeutic principle, offering a solution to the challenge of translating microbiome science into precise pharmacology. This mechanism, integrating direct host‐cell sensitization with targeted microbiome modulation, provides a new blueprint for designing therapies against RIE and potentially other conditions of epithelial barrier injury, such as inflammatory bowel disease and chemotherapy‐induced mucositis. While further validation is required for clinical translation, this work provides a complete, molecule‐to‐organism narrative of how a single therapeutic agent can intelligently co‐opt and coordinate both microbial and host resources to achieve a potent and precise therapeutic outcome, opening new avenues for the development of next‐generation, ecosystem‐targeted therapeutics.

## Author Contributions

Wanjiang Xue and Yilin Hu initially designed this project and conceptualized the research. Jiancheng He, Shukang Deng and Jiapeng Bao conducted the experiments and statistical analyses, and wrote the first draft. Weijie Zang, Haoming Yan,Zihao Zhao carried out the visualizations. Guangze Zhang, Junjie Chen and Ruiqing Liu analyzed the sequencing data. All authors revised and edited the original manuscript.

## Conflicts of Interest

The authors declare no conflicts of interest.

## Supporting information




**Supporting File 1**: advs75867‐sup‐0001‐SIFigures.docx.

**Supporting File 2**: advs75867‐sup‐0002‐SITables.xlsx.

## Data Availability

The data that support the findings of this study are available on request from the corresponding author.
