# Supplementary material for: β‐Elemene Rescues Radiation‐Induced Enteritis by Orchestrating a Host‐Microbiome Circuit That Fuels Epigenetic DNA Repair
Source: Adv Sci (Weinh). 2026 May 27:e21445. Online ahead of print. doi: 10.1002/advs.202521445 (PMC13336028; doi:10.1002/advs.202521445)
Supplement: Supplementary file 1 — Supporting file 1: advs75867‐sup‐0001‐SIFigures.docx. [file ADVS-9999-e21445-s002.docx]

**
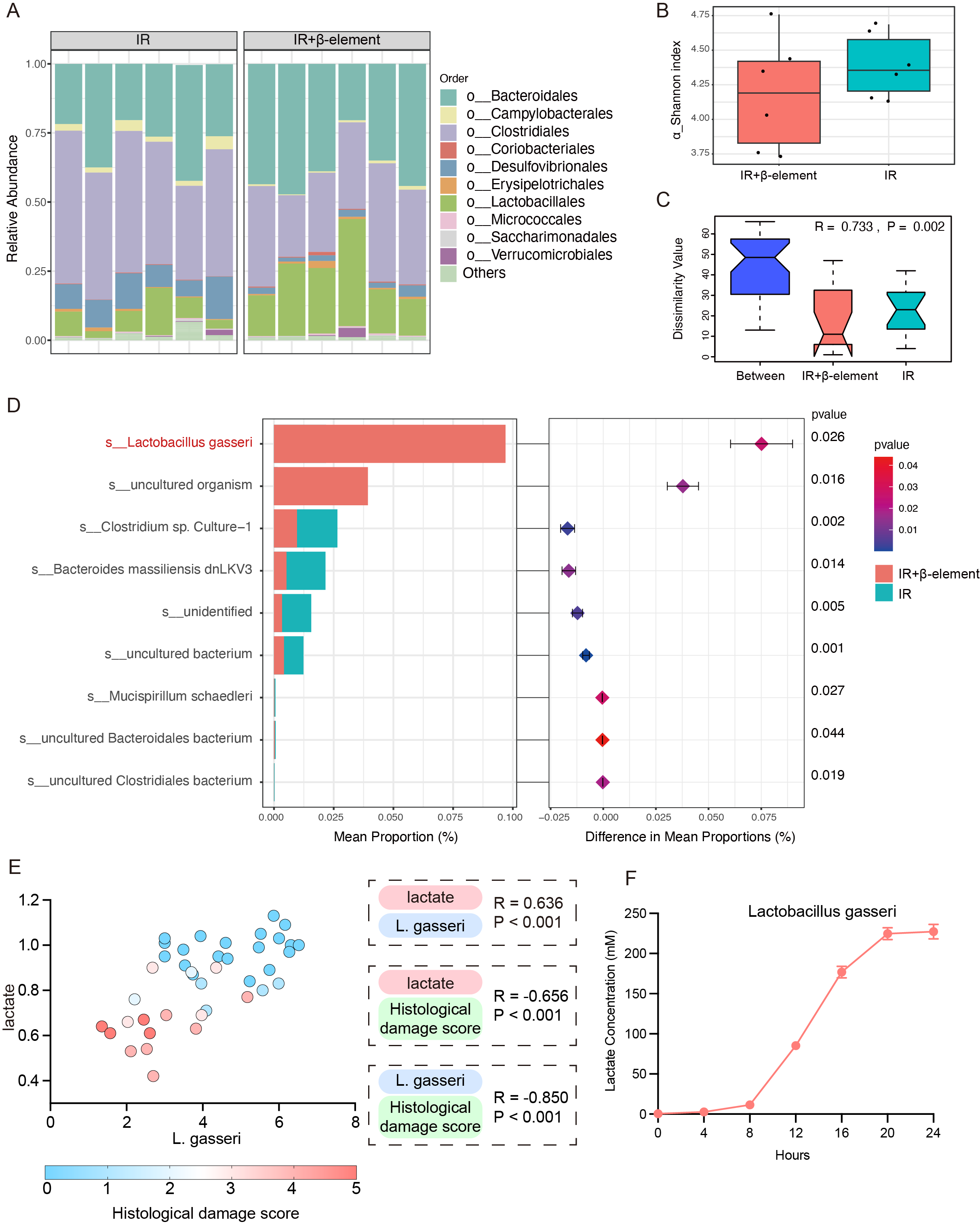
**

**Supplementary Figure 1. β-elemene remodels the gut microbiota composition in irradiated mice.**

(A) Stacked bar chart showing the relative abundance of gut microbiota at the Order level for each mouse in the irradiation-only (IR) and combination treatment (IR+β-elemene) groups. (B) Box plot analysis of α-diversity based on the Shannon Index, comparing the intra-sample microbial diversity between the two groups. (C) Analysis of Similarity (ANOSIM) of inter- and intra-group differences in microbial community structure. The results show a significant difference in microbial composition between the two groups (R = 0.733, P = 0.002). (D) Comparison plot of differential microbial abundance between the two groups at the species level. The left panel shows mean abundance, and the right panel shows an extended error bar plot of the difference in mean proportions. The results clearly indicate that *Lactobacillus gasseri (L. gasseri)* is significantly enriched in the combination treatment group (P = 0.026). (E) Scatter plot showing a significant positive correlation between the relative abundance of *L. gasseri* and lactate concentration (R = 0.636, P < 0.001). Data points are colored according to the histological damage score.(F) In vitro kinetic assay of lactate production by *L. gasseri*. The bacterium was cultured anaerobically, and the lactate concentration in the cell-free culture supernatant was quantified at 0, 4, 8, 12, 16, 20, and 24 hours, demonstrating a robust exponential accumulation that peaked at approximately 20 hours.

**
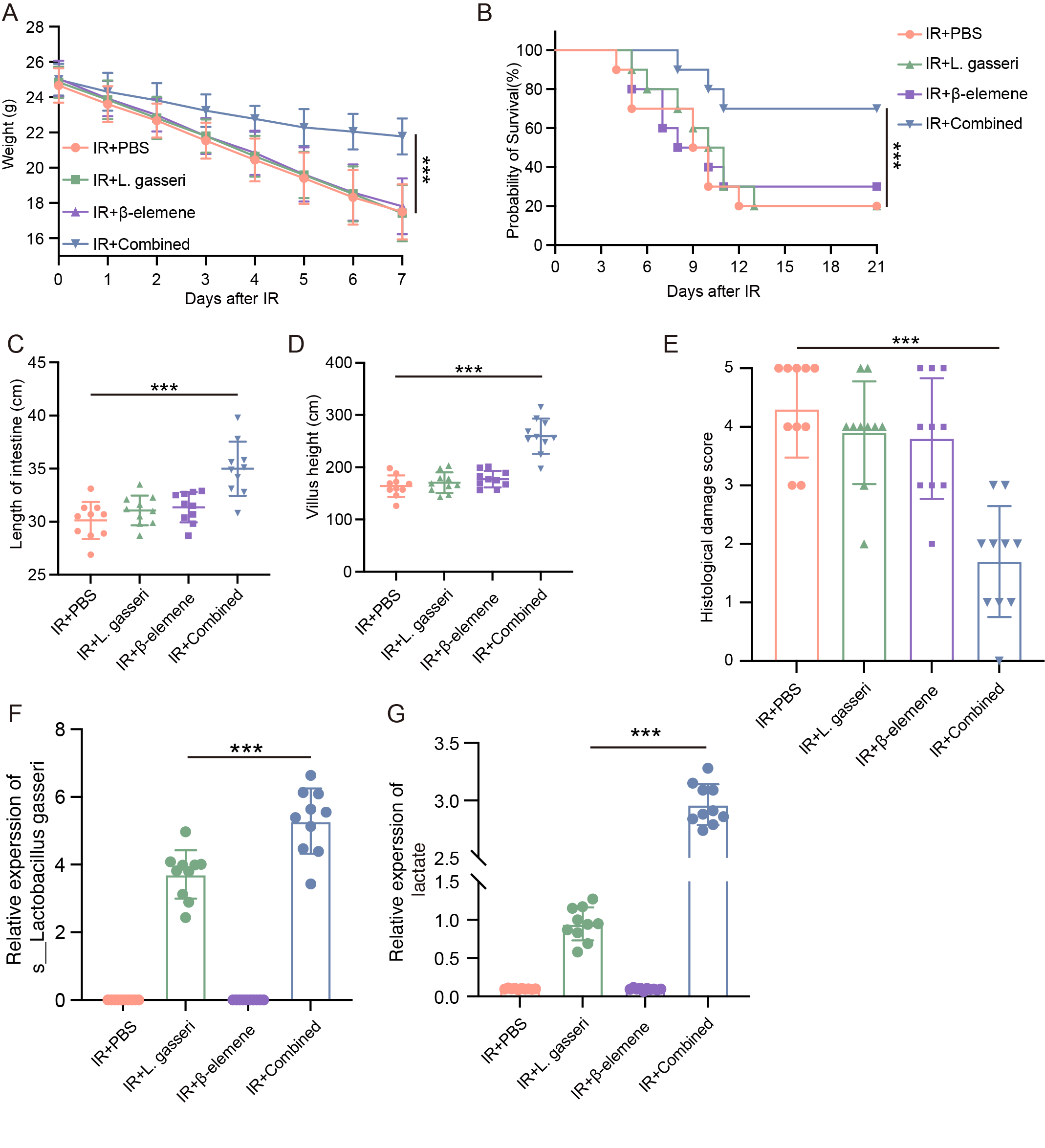
**

**Supplementary Figure 2. *L. gasseri* and β-elemene synergistically protect germ-free mice from radiation-induced intestinal injury.**

(A) Body weight change curves for each group of mice within 7 days after irradiation. (B) Kaplan-Meier survival curves for each group of mice within 21 days after irradiation. (C-E) Quantification of small intestine length (C), villus height (D), and histological damage score (E). (F) Relative abundance of *L. gasseri* in the cecal contents of each group, as determined by RT-qPCR to verify colonization. (G) Relative concentration of lactate in the intestines of each group. All data are presented as mean ± SD. *** P < 0.001.


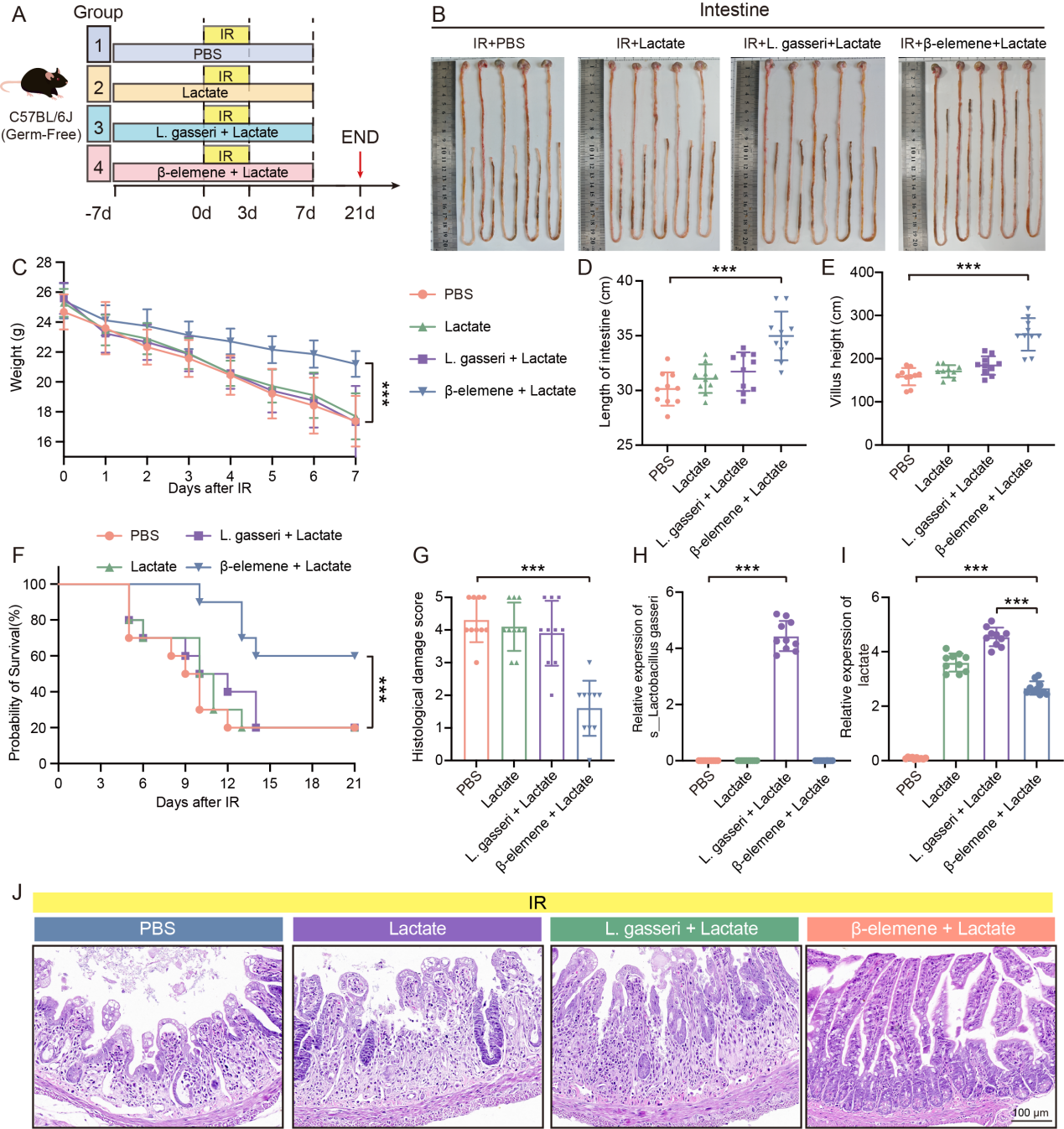


**Supplementary Figure 3. Synergistic radioprotection of GF mice by β-elemene and lactate.**

(A) Experimental schematic for germ-free (GF) C57BL/6J mice. (B–C) Gross intestinal morphology and body weight curves. (D–E) Quantification of intestinal length and villus height. (F–G) Survival analysis and histological damage scores. (H–I) qPCR validation of *L. gasseri* abundance and lactate levels in intestinal contents. (J) Representative H&E-stained ileal sections (Scale bar = 100 μm). Data are presented as mean ± SD; *** P < 0.001.

**
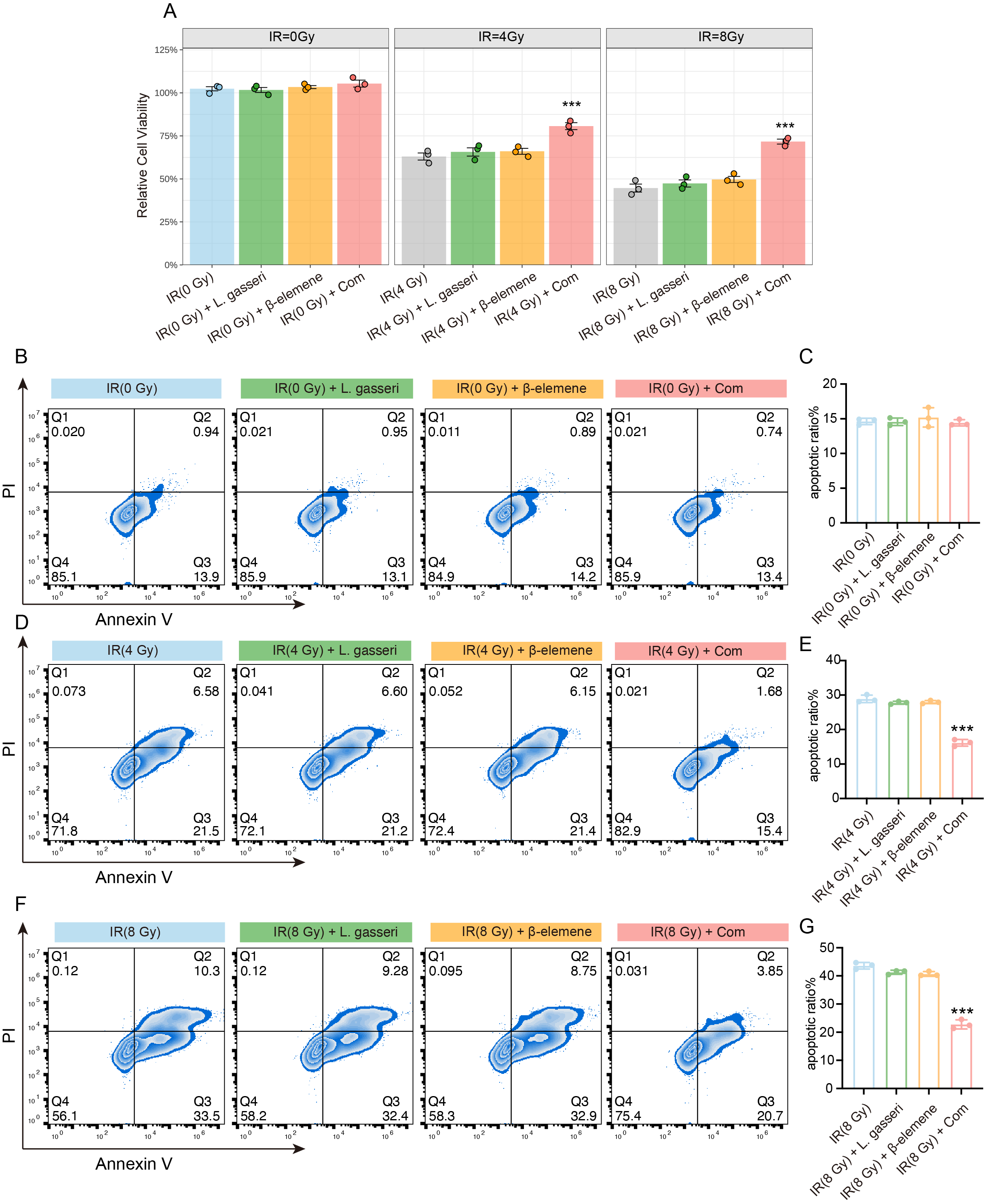
**

**Supplementary Figure 4. Combination treatment with *L. gasseri* supernatant and β-elemene synergistically inhibits radiation-induced apoptosis in HIEC-6 cells.**

(A) The effects of β-elemene and *L. gasseri* supernatant on the relative viability of HIEC-6 cells were assessed following exposure to varying doses of radiation (0, 4, and 8 Gy), including individual and combined treatments (the "Com" group). (B-C) Representative flow cytometry plots (B) and quantification of the apoptotic rate (C) for the 0 Gy irradiation group. (D-E) Representative flow cytometry plots (D) and quantification of the apoptotic rate (E) for the 4 Gy irradiation group. (F-G) Representative flow cytometry plots (F) and quantification of the apoptotic rate (G) for the 8 Gy irradiation group. The results show that, compared to the control groups, only the combination treatment group (IR+Com) could significantly reduce radiation-induced apoptosis. All data are presented as mean ± SD. Statistical significance was assessed by appropriate statistical tests. *** P < 0.001.

**
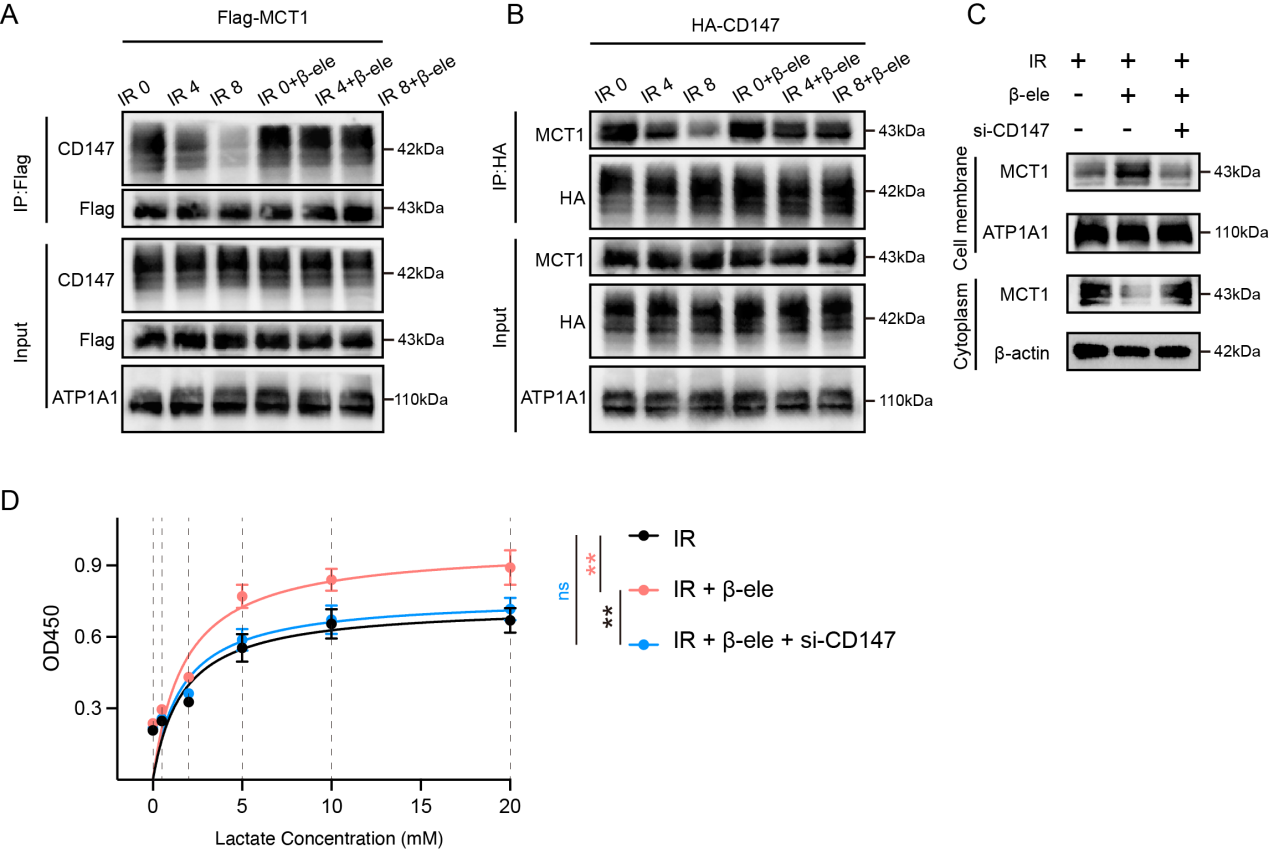
**

**Supplementary Figure 5. β-elemene enhances the interaction between MCT1 and CD147.**

(A) Co-immunoprecipitation experiment in cells overexpressing Flag-MCT1. IP was performed with an anti-Flag antibody, followed by detection of CD147 binding. (B) Co-immunoprecipitation experiment in cells overexpressing HA-CD147. IP was performed with an anti-HA antibody, followed by detection of MCT1 binding. (C) Western blot analysis of fractionated cell components. The results show that β-elemene can promote the localization of MCT1 to the cell membrane after radiation treatment, but this pro-translocation effect is abolished when CD147 is knocked down using siRNA (si-CD147). ATP1A1 and β-actin serve as loading controls for the cell membrane and cytoplasmic fractions, respectively.(D) Intracellular lactate uptake assay in HIEC-6 cells. Cells with or without CD147 knockdown (si-CD147) were treated with irradiation (IR) and/or β-elemene, followed by incubation with varying concentrations of lactate. The intracellular lactate levels (indicated by OD450) demonstrate that the β-elemene-mediated enhancement of lactate uptake post-irradiation is completely abrogated in the absence of CD147. Original full and unaltered blots can be found in Supplementary Figure 12.

**
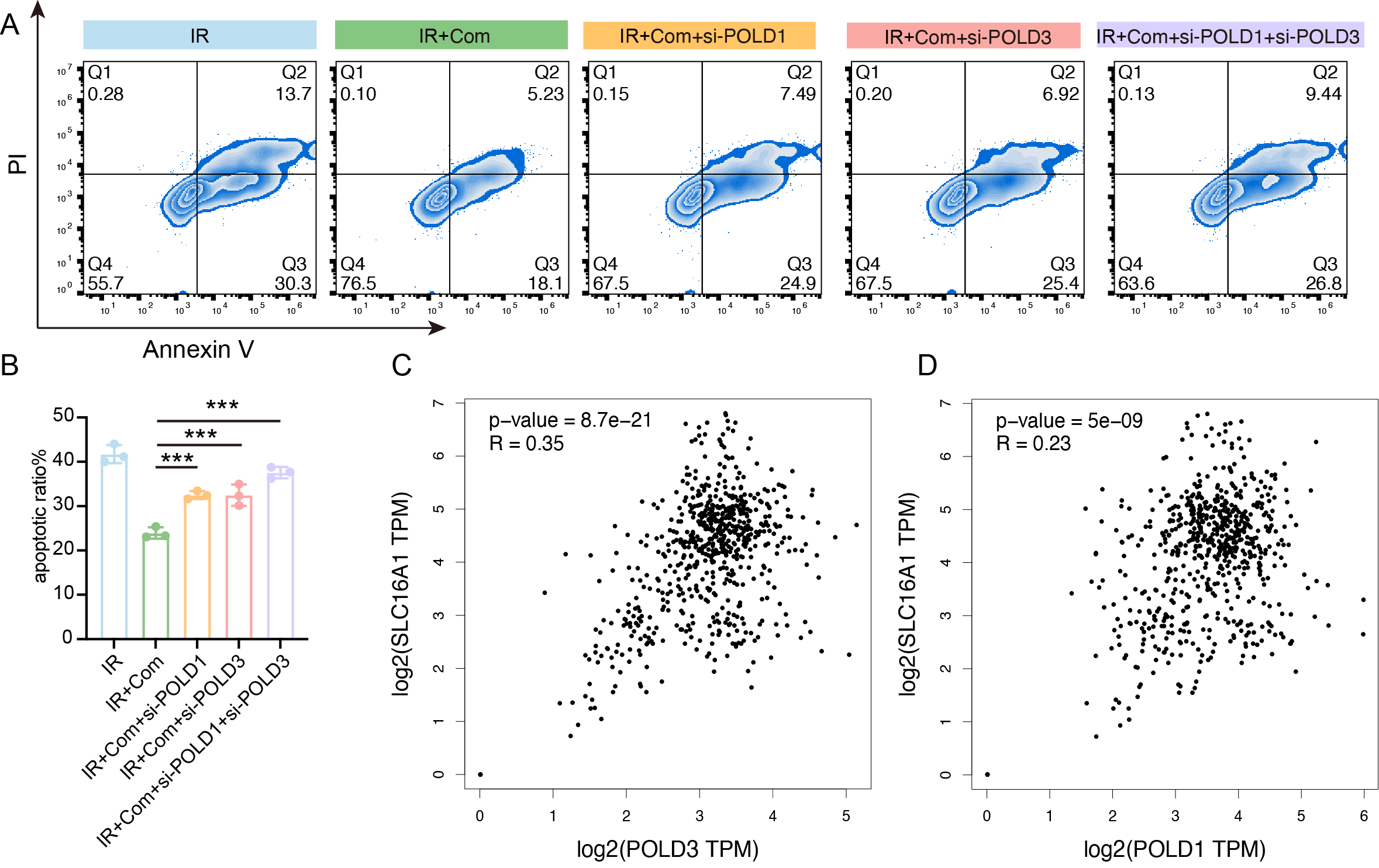
**

**Supplementary Figure 6. The anti-apoptotic effect of combination treatment is dependent on POLD1 and POLD3.**

(A-B) Apoptosis in HIEC-6 cells under the indicated treatment conditions was assessed by Annexin V/PI dual-staining and flow cytometry. Representative flow cytometry plots (A) and quantification of the apoptotic rate (B) are shown. The results indicate that siRNA-mediated knockdown of POLD1, POLD3, or both, significantly reverses the anti-apoptotic protective effect of the combination treatment (IR+Com). (C-D) Gene expression correlation analysis based on the GEPIA2 database. The scatter plots show that the mRNA expression level of the lactate transporter SLC16A1 (MCT1) is significantly positively correlated with the expression levels of both POLD3 (C) and POLD1 (D). All quantitative data are presented as mean ± SD. *** P < 0.001.

**
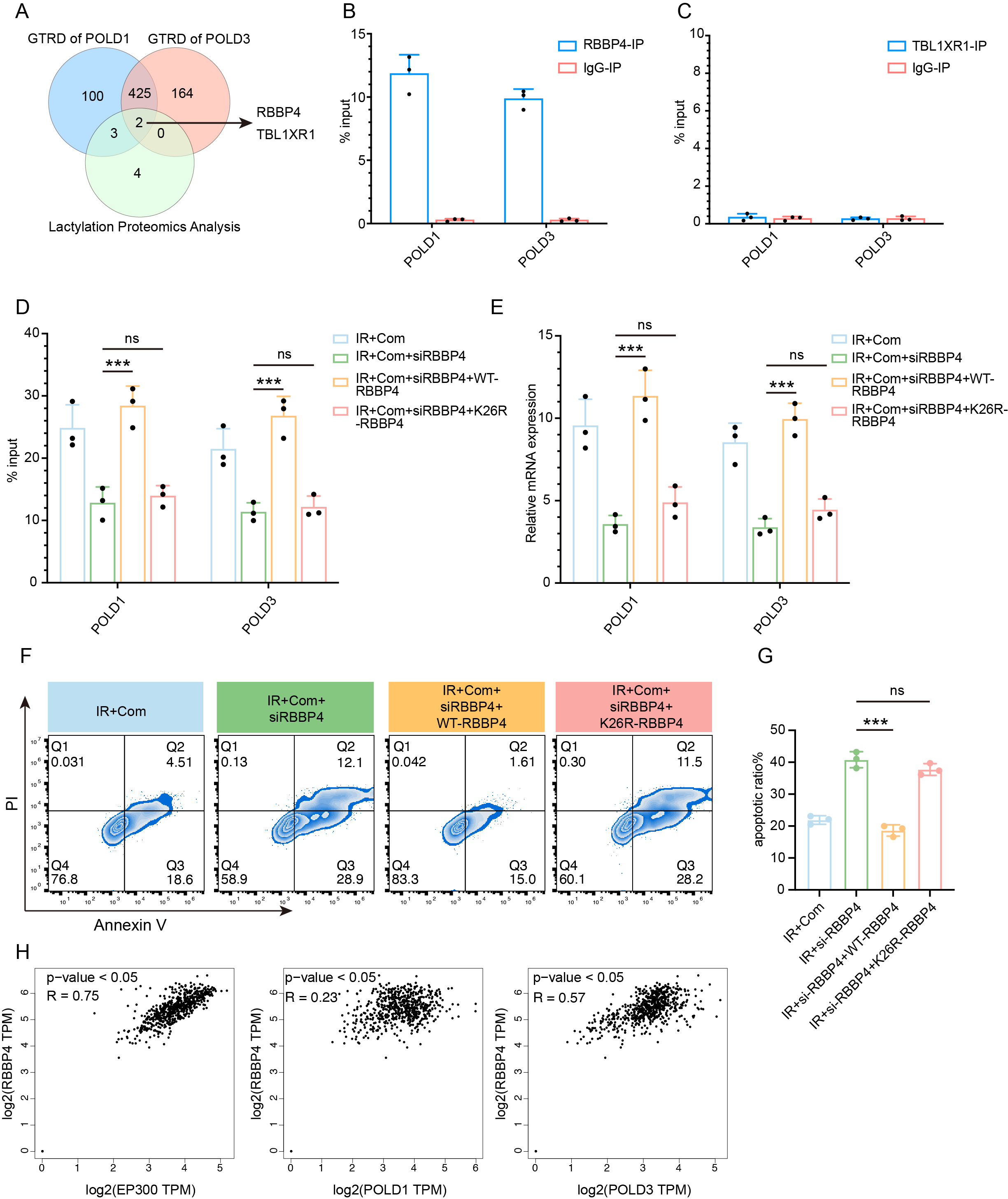
**

**Supplementary Figure 7. Lactylation modification of RBBP4 is crucial for the epigenetic activation of POLD1/3.**

(A) Venn diagram showing the intersection analysis between transcriptional regulators of POLD1 and POLD3 (from the GTRD database) and the lactylated proteome identified in this study, which screened for the two key candidate proteins RBBP4 and TBL1XR1. (B-C) Chromatin immunoprecipitation-quantitative PCR (ChIP-qPCR) analysis. (D) ChIP-qPCR analysis of H3K27ac levels at the POLD1 and POLD3 promoter regions under conditions of RBBP4 knockdown and rescue (with wild-type WT or K26R mutant). (F-G) Representative images (F) and quantification (G) of apoptosis assessed by Annexin V/PI flow cytometry under the same experimental conditions as in (D). (H) Gene expression correlation analysis based on the GEPIA2 database. All quantitative data are presented as mean ± SD. ns, not significant; *** P < 0.001.

**
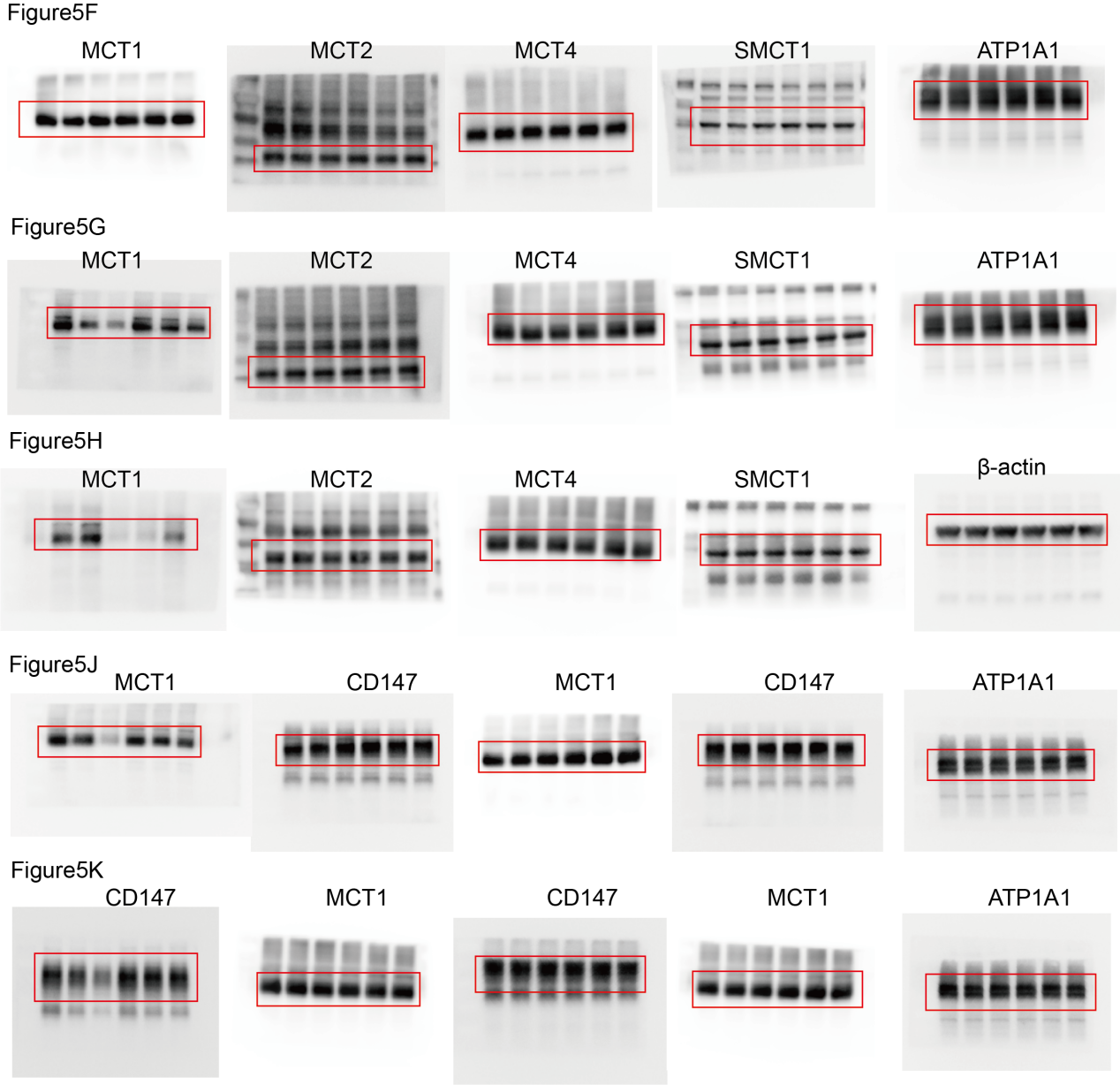
**

**Supplementary Figure 8. Full and unaltered original Western blot images for Figure 5.**

**
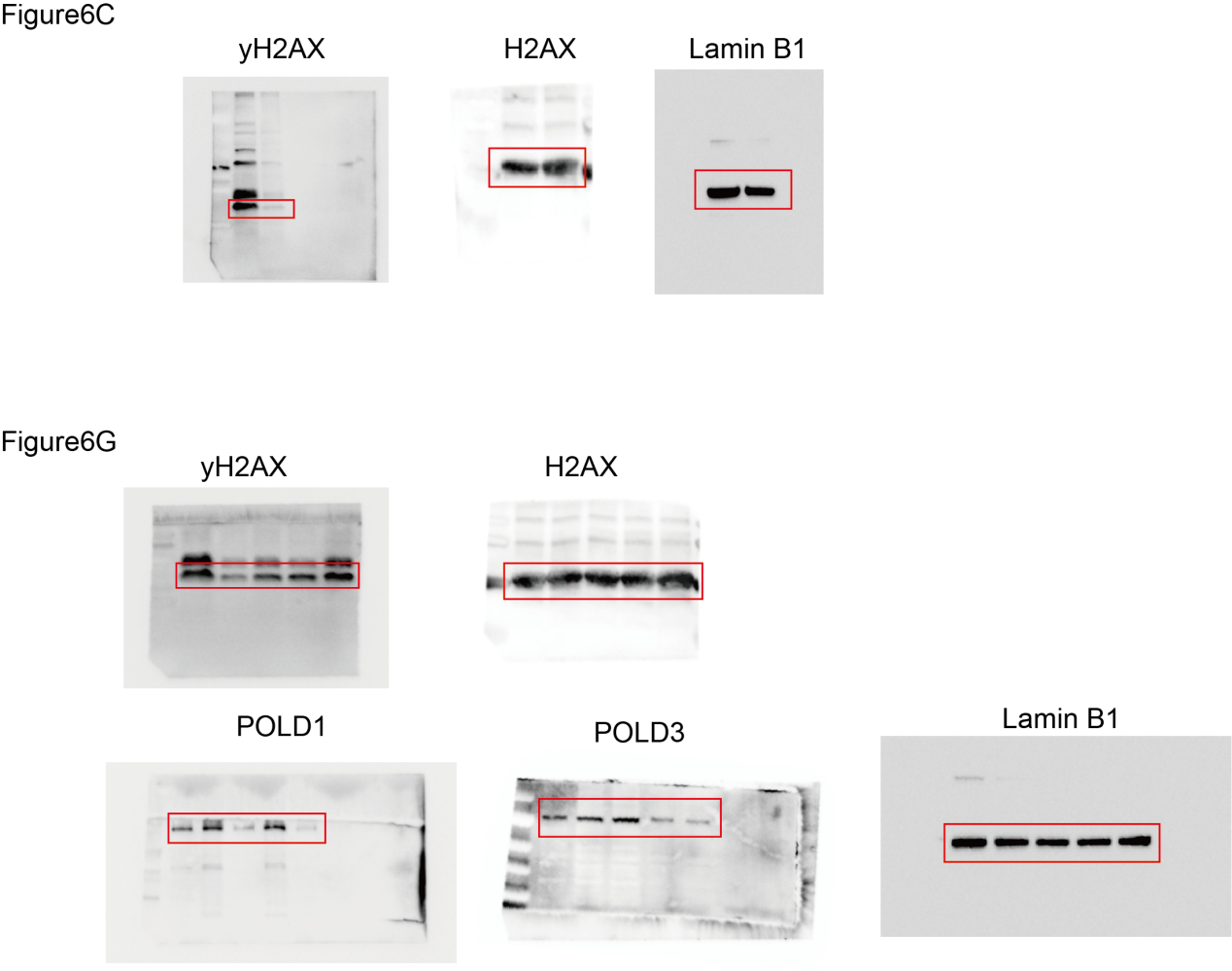
**

**Supplementary Figure 9. Full and unaltered original Western blot images for Figure 6.**

**
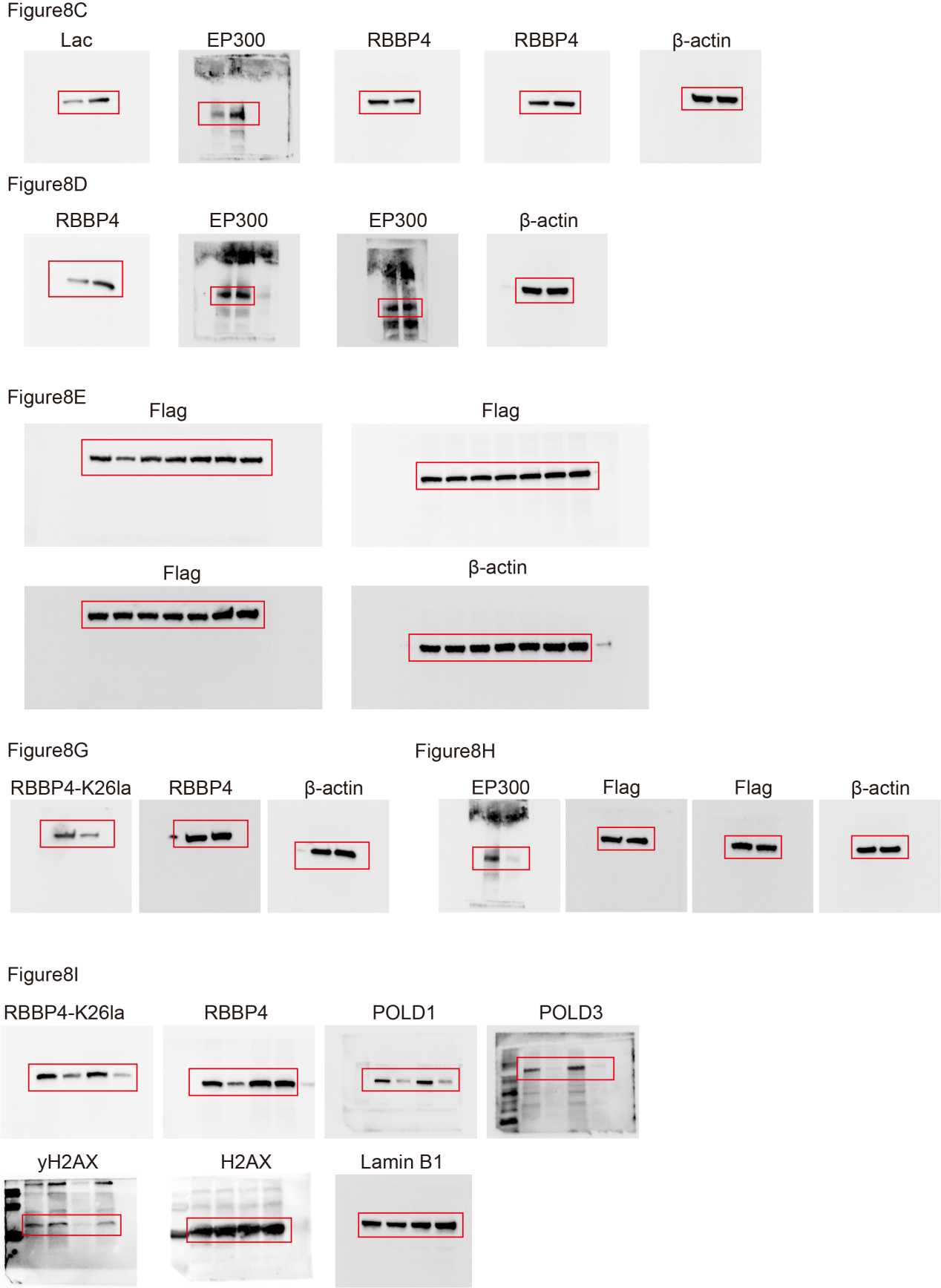
**

**Supplementary Figure 10. Full and unaltered original Western blot images for Figure 8.**

**
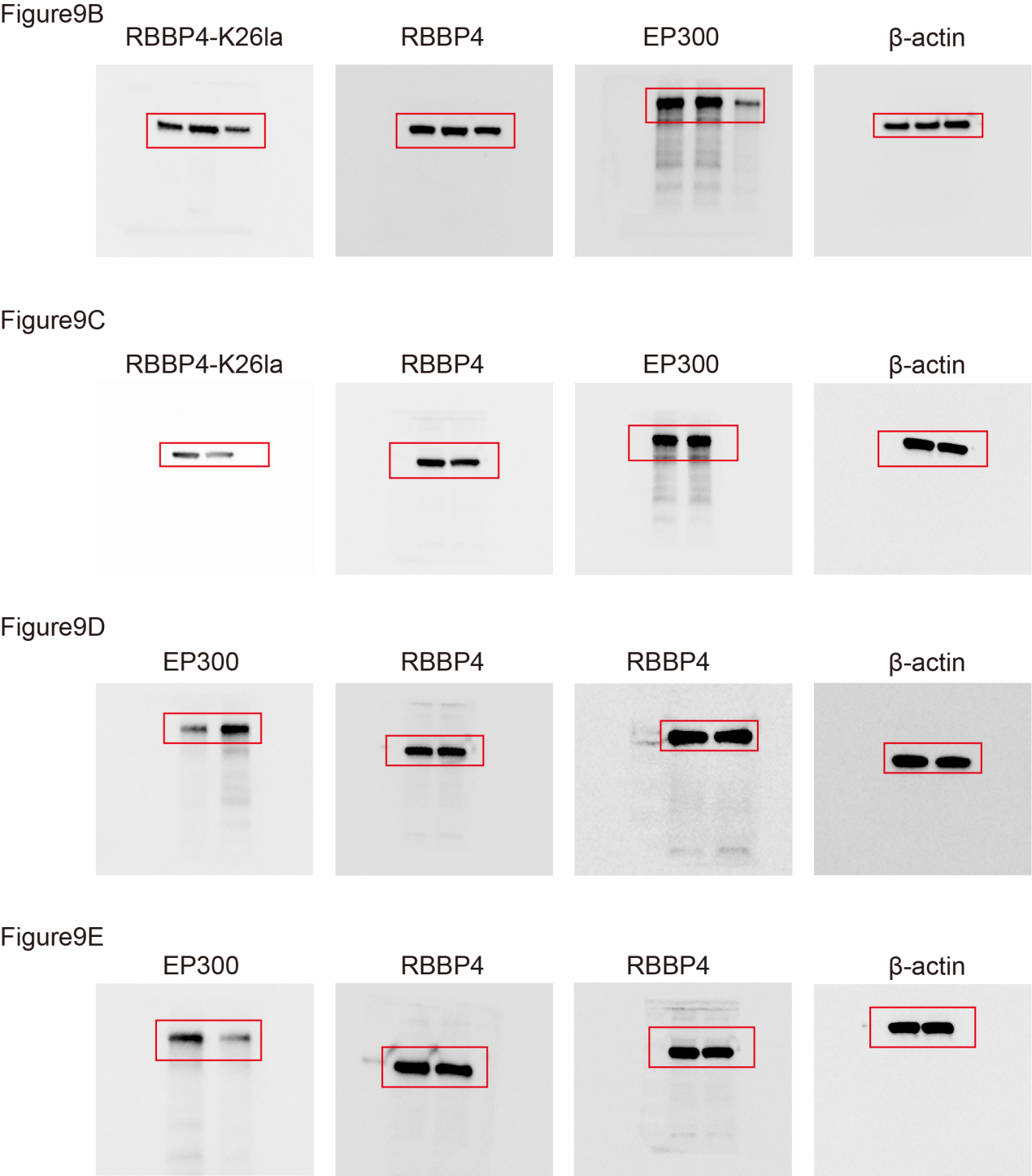
**

**Supplementary Figure 11. Full and unaltered original Western blot images for Figure 9.**

**
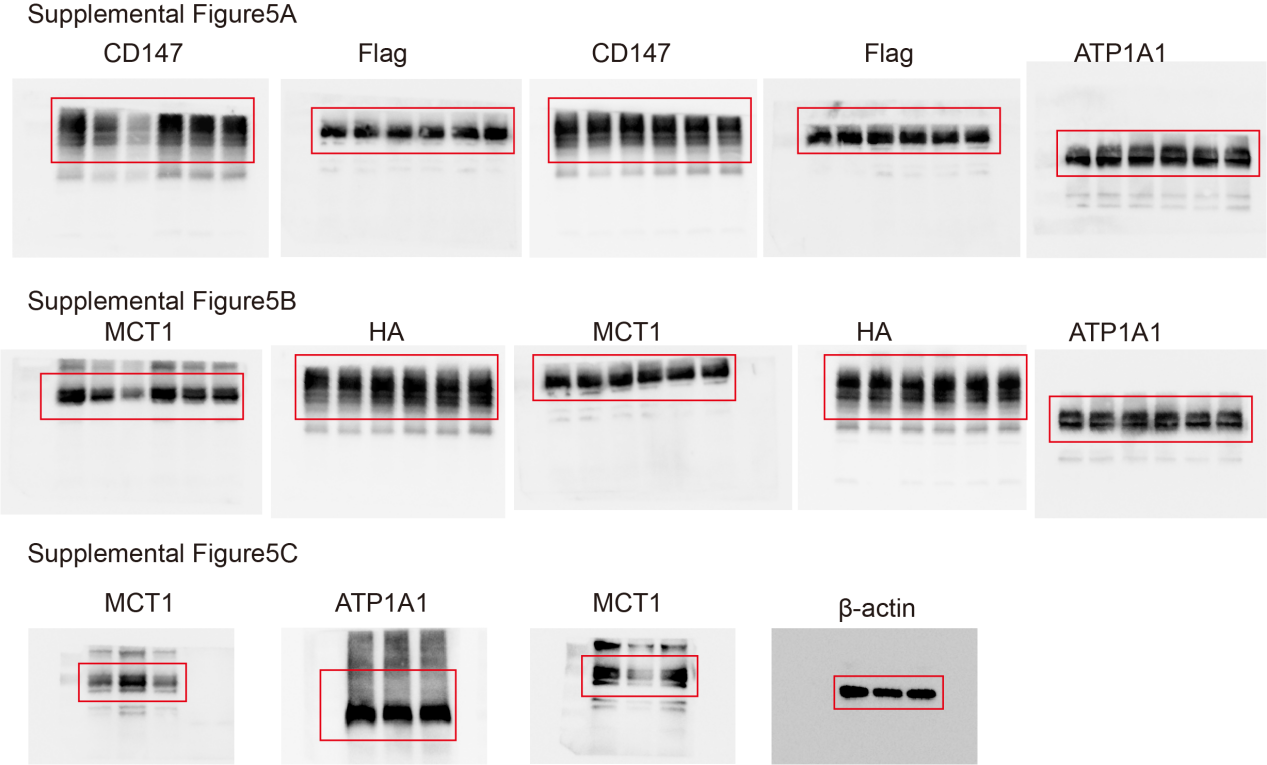
**

**Supplementary Figure 12. Full and unaltered original Western blot images for supplementary Figure 5.**
